# Heat and cause-specific cardiopulmonary mortality in Germany: a case-crossover study using small-area assessment

**DOI:** 10.1016/j.lanepe.2024.101049

**Published:** 2024-09-06

**Authors:** Siqi Zhang, Susanne Breitner, Francesca de' Donato, Massimo Stafoggia, Nikolaos Nikolaou, Kristin Aunan, Annette Peters, Alexandra Schneider

**Affiliations:** a Institute of Epidemiology, Helmholtz Zentrum München, Neuherberg, Germany; b Department of Environmental Health Sciences, Yale School of Public Health, New Haven, United States; c Institute for Medical Information Processing, Biometry, and Epidemiology, Faculty of Medicine, LMU, Munich, Germany; d Department of Epidemiology, Lazio Regional Health Service - ASL ROMA 1, Rome, Italy; e CICERO Center for International Climate Research, Norway; f Centre for Cardiovascular Research (DZHK), Partner Site Munich Heart Alliance, Munich, Germany

**Keywords:** Temperature, Cardiovascular disease, Respiratory disease, Vulnerability factor, Urbanization, Air pollution, Greenness

## Abstract

**Background:**

High temperatures have been associated with increased mortality, with evidence reported predominately in large cities and for total cardiovascular or respiratory deaths. This case-crossover study examined heat-related cause-specific cardiopulmonary mortality and vulnerability factors using small-area data from Germany.

**Methods:**

We analyzed daily counts of cause-specific cardiopulmonary deaths from 380 German districts (2000–2016) and daily mean temperatures estimated by spatial–temporal models. We applied conditional quasi-Poisson regression using distributed lag nonlinear models to examine heat effects during May–September in each district and random-effects meta-analysis to pool the district-specific estimates. Potential individual- and district-level vulnerability factors were examined by subgroup analyses and meta-regressions, respectively.

**Findings:**

Heat was associated with increased mortality risks for all cardiopulmonary sub-causes. The relative risk (RR) of total cardiovascular and respiratory mortality for a temperature increment from the 75th to the 99th percentile was 1.24 (95% confidence interval: 1.23, 1.26) and 1.34 (1.30, 1.38), respectively. The RRs of cardiovascular sub-causes ranged from 1.16 (1.13, 1.19) for myocardial infarction to 1.32 (1.29, 1.36) for heart failure. For respiratory sub-causes, the RR was 1.27 (1.22, 1.31) for COPD and 1.49 (1.42, 1.57) for pneumonia. We observed greater susceptibility related to several individual- and district-level characteristics, e.g., among females or in highly urbanized districts. Heat vulnerability factors remained consistent between urban and rural areas.

**Interpretation:**

Our study highlights heat-related increases in cause-specific cardiopulmonary mortality across Germany and identifies key vulnerability factors, offering insights for improving public health practices to mitigate heat-related health impacts.

**Funding:**

European Union's 10.13039/501100007601Horizon 2020 research and innovation program; Helmholtz Associations Initiative and Networking Fund.

**Research in context**

**Evidence before this study**

We searched PubMed and Google Scholar for relevant studies published up to April 24, 2024. The search terms used were (“temperature” OR “heat” OR “climate change”) AND (“cardiorespiratory” OR “cardiovascular” OR “respiratory” OR “heart” OR “lung” OR “pulmonary” OR “ischemic heart disease” OR “myocardial infarction” OR “heart failure” OR “cerebrovascular” OR “stroke” OR “chronic obstructive pulmonary disease” OR “pneumonia”) AND (“mortality” OR “death”). While numerous studies have established associations between heat and overall cardiovascular or respiratory mortality, few have specifically examined the impact of heat on different sub-causes of cardiorespiratory deaths. Moreover, our search identified only 17 nationwide or regional studies that analyzed mortality data from both urban and rural settings, including eight from Asia, seven from Europe, and one each from South America and Africa. The majority of studies were confined to major cities or urban areas, which may have restricted the generalizability of their findings due to the distinct characteristics between urban and rural areas.

**Added value of this study**

To the best of our knowledge, this is the first nationwide study on heat and cause-specific cardiopulmonary mortality and related vulnerability factors in Germany. Our study found heat-related increased risks for various causes of cardiorespiratory mortality during the warm season, with the strongest effects on heart failure and pneumonia mortality. Greater susceptibility to heat was observed among females and in districts with higher degrees of urbanization, elevated levels of PM_2.5_ or NO_2_, or higher mean air temperatures over the study period. Heat vulnerability factors varied across sub-causes of deaths but remained consistent between urban and rural regions.

**Implications of all the available evidence**

Our study contributes to the existing evidence on the association between heat and cause-specific cardiorespiratory mortality. By identifying vulnerable subgroups and areas, our findings may inform policymakers and public health practitioners of the people most at risk, thereby prioritizing resources and interventions effectively in the face of rising temperatures and climate change. Moreover, the heat effect modification by modifiable environmental factors emphasizes the importance of implementing stringent air quality regulations and enhancing green infrastructure as crucial strategies to mitigate heat-related health risks and build resilience to heat within communities.

## Introduction

Cardiovascular diseases (CVD) and respiratory diseases (RD) are leading causes of death worldwide. The Global Burden of Disease study estimated that CVD and chronic RD accounted for approximately 18.6 million and 4.0 million deaths, respectively, in 2019.^1^ Notably, ischemic heart disease (IHD), stroke, and chronic obstructive pulmonary disease (COPD) ranked as the top three causes of death globally among all conditions.^1^ These findings highlight the significant impacts of CVD and RD on public health systems. Hence, identifying risk factors for these diseases is imperative for effective public health interventions.

Thermoregulatory responses to heat stress, including vasodilation, excessive sweating, hyperventilation, and impaired autonomic nervous system control, may induce cardiovascular strain and increased pulmonary stress.^2^ These physiological changes could exacerbate pre-existing conditions and contribute to severe cardiopulmonary events. Numerous epidemiological studies have demonstrated an association between heat exposure and increased mortality risks from CVD and RD.^3^^,^^4^ Nevertheless, few studies have systematically evaluated the heat impact on cause-specific cardiopulmonary mortality, particularly in Europe. With the global rise in temperatures due to climate change, the heat-related excess mortality is projected to be further amplified, posing a significant threat to susceptible populations.^5^

The impact of heat on mortality varies across geographical regions, which could be attributable to diverse sociodemographic, environmental, and climatic characteristics. Previous studies have reported associations of heat vulnerability with population density, gross domestic product (GDP), income inequalities, air pollution, and green spaces.^6^, ^7^, ^8^ However, evidence of the area-level heat vulnerability factors is still limited, especially for cause-specific cardiopulmonary mortality.^9^ In addition, existing studies have been mostly confined to cities, thereby limiting the generalizability of their findings considering the distinct characteristics between urban and rural areas.

This study assessed associations between heat exposure and cause-specific cardiopulmonary mortality during the warm season (May–September) using data from both urban and rural districts across Germany. We further investigated heat effect modifications by individual- and district-level characteristics to identify factors related to heat vulnerability for different mortality endpoints. Furthermore, we evaluated potential disparities in the heat vulnerability factors between urban and rural contexts.

## Methods

### Data sources

We obtained daily mortality data across Germany from the Research Data Centre of the Federal Statistical Office and Statistical Offices of the Federal States for 2000–2016. The original mortality data were at the individual level, including information on the cause of death, age at death, sex, and district of residence. Districts are administrative subdivisions in Germany with an average population of around 215,000 during the study period. Death causes were classified in accordance with the International Classification of Diseases, Tenth Revision (ICD-10). Causes analyzed in the current study comprised cardiovascular diseases (CVD, ICD-10: I00–I99), ischemic heart diseases (IHD, ICD-10: I20–I25), myocardial infarction (MI, I21) as a subtype of IHD, heart failure (HF, I50), cerebrovascular diseases (I60–I69), respiratory diseases (RD, J00–J99), chronic obstructive pulmonary diseases (COPD, J40–J44 and J47), and pneumonia (J12–J18). Daily counts of deaths from the specified causes were aggregated at the district level for the entire population as well as specific age groups (65+ and 75+ years) and sex (males and females).

Gridded daily mean air temperatures in Germany at a spatial resolution of 1 × 1 km were generated using a 3-stage regression-based modeling approach.^10^ The model incorporated air temperature observations from weather stations (T_air_), satellite-based land surface temperature (LST), and spatial predictors, including elevation, vegetation, and multiple land use predictors (percentages of urbanized areas, arable land, pastures, forests, and water bodies). In the first stage, for grid cells with both T_air_ and LST data available, T_air_ was regressed against LST by a linear mixed-effects model with spatial predictors and daily random intercepts and slopes for LST. In the second stage, the first-stage model was used to predict T_air_ in grid cells with only daily LST data. In the third stage, for grid cells and days having neither T_air_ nor LST, the T_air_ predictions from the second stage were regressed against T_air_ values interpolated by a thin plate spline technique to obtain fully spatiotemporally covered air temperature data. All models achieved high accuracy (0.91 ≤ R^2^ ≤ 0.98) and low errors (Root Mean Square Error ≤2.02 °C) in the 10-fold cross-validation, indicating good model performance. We estimated daily mean air temperatures in each district by calculating area-weighted averages within grids that intersected with respective districts. The assignment of weights was based on the degree of overlap between the grid cells and the district boundary. The exposure assessment generated a time series of daily mean temperatures for each district during the study period from 2000 to 2016. In the main analysis, we used same-day and previous-day temperatures to emphasize the acute effects of heat since previous evidence suggested the strongest association between heat and mortality at lag 0–1 days.^11^

We collected district-level characteristics from the INKAR database (https://www.inkar.de) of the Federal Office for Building and Regional Planning (BBSR) and the Regional Atlas database (https://regionalatlas.statistikportal.de) provided by the German Federal Statistical Office. The investigated characteristics included population structure (percentage of individuals aged ≥65 years and proportion of foreigners), socio-economic status (unemployment rate and GDP per capita in Euro), population and housing density (population density and living space per capita), and type of district (urban or rural, districts with a population density of at least 150 inhabitants/km^2^ were defined as urban districts). For each district, we computed the mean values of these characteristics by aggregating data from all available years within the study period. Additionally, land use patterns, including coverages of urbanized areas, green areas, and water bodies, were acquired from the Corine Land Cover dataset (https://land.copernicus.eu/pan-european/corine-land-cover) for the year 2012. Daily mean concentrations of fine particulate matter (PM_2.5_), ozone (O_3_), and nitrogen dioxide (NO_2_) were estimated at an approximate spatial resolution of 2 × 2 km for the years 2004–2016 using a spatiotemporal model based on optimal interpolation.^12^ Long-term air pollution exposure at the district level was assessed by averaging the daily mean air pollutant concentrations over the available years. Similarly, we determined the district-specific mean temperature over the study period by averaging the whole-year daily mean air temperatures from 2000 to 2016.

### Statistical analysis

We applied a case-crossover study design with the conditional quasi-Poisson regression model in each district to investigate associations between heat exposure and cause-specific cardiopulmonary mortality in the warm season (May–September).^13^ A distributed lag non-linear model (DLNM) was employed to define the relationship between temperature and mortality while accounting for the lagged effects. The exposure-response function was specified using a B-spline with an internal knot at the 50th percentile of the district-specific temperature distribution. This knot choice allows for flexibility in capturing potentially non-linear relationships between temperature and mortality across the range of warm-season temperatures. The lag structure in the main analysis was restricted to 0–1 days to focus on the acute effects of heat. Given the short lag period, we used a simple linear function to model the lagged effects. The conditional quasi-Poisson regression model was adjusted for a three-way interaction between the year, month, and day of the week to control for the district-specific time trend.^14^ This approach is equivalent to the case-crossover design using the conditional logistic regression and is able to account for overdispersion.^13^^,^^14^ We then retrieved the regression coefficients that captured the cumulative exposure-response function in each district. These coefficients were synthesized in a multivariate meta-analysis to derive the overall relationship between temperature and cause-specific cardiopulmonary mortality across Germany. We estimated heat effects as the relative risks (RRs) with 95% confidence intervals (95% CIs) for an increase in the daily mean temperature from the 75th to the 99th percentile of the German-wide warm season distribution. The RR was calculated by exponentiating the difference in the temperature effects predicted at the 99th and 75th percentiles of the temperature distribution, representing the relative change in mortality risk associated with increased temperature. We used an increment from the 75th to the 99th percentile of the temperature distribution when presenting heat effects to focus on the higher end of the temperature spectrum, which is most relevant for assessing the impact of extreme heat on mortality. The selection of these percentiles is consistent with previous studies examining the impact of high temperatures on health outcomes,^15^^,^^16^ providing a robust basis for understanding the public health implications of extreme heat. This analytical procedure was performed on daily death counts in the entire population, as well as in different age (over 65 or 75 years) and sex (males and females) groups to examine the effect modification by these two individual-level characteristics. The statistical significance of between-group differences was assessed by the two-sided Z-test.^17^

The potential heat effect modification by area characteristics was assessed using multivariate mixed-effects meta-regression with a random effect of districts. The meta-regression incorporated the district-specific conditional quasi-Poisson regression coefficients as the dependent variable and each district-level characteristic as the explanatory variable (single-predictor model). We evaluated the statistical significance of the effect modification using the Wald test.^18^ To account for the potential influence of urbanization on the other characteristics, we built two-predictor meta-regression models, adjusting for the percentage of urbanized areas for characteristics that were not highly correlated with urbanization (absolute values of Spearman correlation coefficients <0.7). RRs (95% CIs) associated with an increase in temperature from the 75th to the 99th percentile were estimated at low and high levels of continuous characteristics, defined respectively as the 25th and 75th percentiles of the district-specific characteristic distribution. Moreover, to explore the potential differences in effect modification between urban and rural areas, we additionally built two-predictor models including the type of district (urban or rural) and each characteristic that significantly modified the heat effects on CVD or RD mortality. We then estimated the heat effects at low (25th percentile) and high (75th percentile) levels of characteristics separately for urban and rural districts as percent changes in the mortality risk for a temperature increment from the 75th to the 99th percentile.

We conducted several sensitivity analyses to test the robustness of our main findings. We first restricted the warm season to June–August, representing Germany's hottest three months. Second, we extended the lag period of heat effects in DLNM to three days, aiming to incorporate potential delayed effects. Third, we estimated the heat effects for an increase in temperature from the minimum mortality temperature (MMT) to the 99th percentile. Fourth, we further adjusted the quasi-Poisson regression models for the moving averages of individual air pollutants at lag 0–1 days, including PM_2.5_, O_3_, and NO_2_.

All analyses were performed using the R software (version 4.2.1) with the “gnm”, “dlnm”, and “mixmeta” packages.

### Role of the funding source

The funder of the study had no role in study design, the collection, analysis, or interpretation of data, the writing of the report, or the decision to submit the paper for publication. Multiple authors had full access to all the data in the study and the corresponding author had final responsibility for the decision to submit for publication.

## Results

### Descriptions

During the warm season of the study period (2000–2016), 2,050,764 CVD deaths and 299,249 RD deaths were documented in 380 districts across Germany (Table 1, Supplementary Table S1). The largest proportion of CVD deaths were attributed to IHD (38.8%), followed by cerebrovascular diseases (17.6%) and HF (14.2%). COPD and pneumonia accounted for 45.9% and 33.1% of RD deaths, respectively.

**Table 1:** Descriptive statistics of district-specific cardiovascular and respiratory deaths from May to September of the study period (2000–2016).

Outcome	N. events (%)^a^	Daily mean ± SD
**CVD**	2,050,764 (100.0)	2.07 ± 2.71
IHD	796,360 (38.8)	0.81 ± 1.29
MI	315,629 (15.4)	0.32 ± 0.66
HF	290,553 (14.2)	0.29 ± 0.65
Cerebrovascular	360,864 (17.6)	0.37 ± 0.72
**RD**	299,249 (100.0)	0.30 ± 0.68
COPD	137,251 (45.9)	0.14 ± 0.42
Pneumonia	99,131 (33.1)	0.10 ± 0.35

COPD, chronic obstructive pulmonary disease; CVD, cardiovascular disease; HF, heart failure; IHD, ischemic heart disease; MI, myocardial infarction; RD, respiratory disease; SD, standard deviation.

a Percentage of cause-specific CVD and RD deaths relative to the total number of CVD and RD deaths, respectively.

The district-specific daily mean air temperature from May to September ranged from 0.5 °C to 31.0 °C, with a mean of 15.7 °C (Supplementary Table S2). Air temperature was positively correlated with daily mean concentrations of O_3_ (Spearman correlation coefficient *r* = 0.52), and negatively associated with NO_2_ (*r* = −0.44).

The distributions of district characteristics are summarized in Table 2. We observed strong positive correlations between population density, the percentage of urbanized areas, and long-term exposure levels of PM_2.5_ and NO_2_ (*r* > 0.70), and these characteristics were negatively correlated with O_3_ concentrations (−0.77 ≤ *r* ≤ −0.53, Supplementary Table S3). Besides, population density was positively associated with the proportion of foreigners (*r* = 0.72).

**Table 2:** Distribution of district-level characteristics during the study period (2000–2016).

Characteristic	Min.	P5	P25	Median	P75	P95	Max.
Population aged ≥65 years (%)	14.3	17.0	18.7	19.8	21.2	23.7	25.6
Foreigners (%)	1.0	1.7	4.4	6.8	10.5	15.8	28.4
Unemployment rate (%)	2.6	3.7	5.1	7.0	9.9	15.1	19.7
GDP per capita (€1000)	12.7	17.4	21.6	25.5	31.5	53.2	105.2
Population density (persons/km^2^)	39	77	123	210	675	2051	4357
Living space per capita (m^2^)	35.9	38.6	41.2	44.4	47.0	50.1	59.3
Urbanized area (%)	2.0	2.9	4.9	7.0	15.8	35.3	57.8
Green areas (km^2^/100,000 persons)	6.9	22.7	119.4	453.8	781.9	1305.5	2602.1
Water bodies (km^2^/100,000 persons)	0.0	0.0	0.4	1.7	5.8	21.5	144.6
PM_2.5_ (μg/m^3^)^a^	8.1	10.0	11.4	11.9	13.1	15.5	18.4
O_3_ (μg/m^3^)^a^	34.3	38.4	43.9	47.5	51.1	56.4	63.5
NO_2_ (μg/m^3^)^a^	5.8	8.6	11.2	13.7	18.0	26.9	33.9
Temperature (°C)^b^	6.3	7.9	8.9	9.5	10.0	10.7	11.1

GDP, gross domestic product; NO_2_, nitrogen dioxide; O_3_, ozone; PM_2.5_, particulate matter with a diameter of 2.5 μm or less.

a Long-term air pollutant averages were calculated based on the whole-year daily mean air pollutant concentrations from 2004 to 2016.

b Mean temperature during the study period was calculated based on the whole-year daily mean temperatures from 2000 to 2016.

Compared to rural areas, urban areas generally had higher proportions of foreigners, GDP per capita, population density, long-term exposure levels of PM_2.5_ and NO_2_, and annual mean temperature, but lower levels of living space per capita, green areas, water bodies, and O_3_ concentrations (Supplementary Table S4).

### Heat effects on cause-specific cardiopulmonary mortality

The exposure-response functions between air temperature and all examined cardiopulmonary death causes showed J-shape curves, with steeper increases as the air temperature rose (Supplementary Figure S1). An increase in temperature from the 75th to the 99th percentile was associated with pooled RRs of 1.24 (95% CI: 1.23, 1.26) for CVD mortality and 1.34 (95% CI: 1.30, 1.38) for RD mortality (Table 3). The heat effect estimates for cause-specific CVD mortality ranged from 1.16 (95% CI: 1.13, 1.19) for MI to 1.32 (95% CI: 1.29, 1.36) for HF. We observed the most substantial heat impact on pneumonia mortality, with a RR of 1.49 (95% CI: 1.42, 1.57).

**Table 3:** Pooled relative risks (95% confidence intervals) of cause-specific cardiopulmonary mortality across Germany for an increase in air temperature from the 75th to the 99th percentile.

Mortality	Main	Age ≥65 y	Age ≥75 y	Males	Females
**CVD**	1.24 (1.23, 1.26)	1.25 (1.23, 1.26)	1.26 (1.24, 1.28)	1.22 (1.20, 1.24)	1.26 (1.24, 1.27)
IHD	1.21 (1.19, 1.23)	1.22 (1.20, 1.24)	1.23 (1.21, 1.26)	1.18 (1.15, 1.21)	1.24 (1.21, 1.27)
MI	1.16 (1.13, 1.19)	1.17 (1.14, 1.20)	1.19 (1.16, 1.23)	1.12 (1.08, 1.16)	1.21 (1.17, 1.25)
HF	1.32 (1.29, 1.36)	1.32 (1.29, 1.36)	1.33 (1.29, 1.37)	1.31 (1.25, 1.37)	1.33 (1.29, 1.38)
Cerebrovascular	1.25 (1.23, 1.28)	1.25 (1.23, 1.28)	1.26 (1.22, 1.29)	1.29 (1.24, 1.34)	1.24 (1.20, 1.27)
**RD**	1.34 (1.30, 1.38)	1.35 (1.31, 1.39)	1.38 (1.34, 1.42)	1.30 (1.26, 1.35)	1.38 (1.32, 1.44)
COPD	1.27 (1.22, 1.31)	1.26 (1.22, 1.31)	1.30 (1.24, 1.36)	1.25 (1.19, 1.31)	1.31 (1.24, 1.39)
Pneumonia	1.49 (1.42, 1.57)	1.51 (1.44, 1.59)	1.54 (1.47, 1.62)	1.44 (1.34, 1.55)	1.55 (1.45, 1.65)

Conditional quasi-Poisson regression model with adjustment for a three-way interaction between the year, month, and day of the week was used to estimate the district-specific regression coefficients for a temperature increase from the 75th to the 99th percentile. Multivariate meta-analysis was then employed to derive the overall estimate across districts.

COPD, chronic obstructive pulmonary disease; CVD, cardiovascular disease; HF, heart failure; IHD, ischemic heart disease; MI, myocardial infarction; RD, respiratory disease.

In subgroup analyses, the heat effects were slightly more pronounced among people aged 75 years and above, but the differences compared to the entire study population were not statistically significant (Table 3, Supplementary Table S5). Moreover, significantly stronger heat effects were found in females for CVD, IHD, MI, and RD mortality. Such higher susceptibility of females remained when sex-stratified analyses were conducted in the same age subgroup (Supplementary Table S6).

### Heat effect modification by area characteristics

**Fig. 1:**
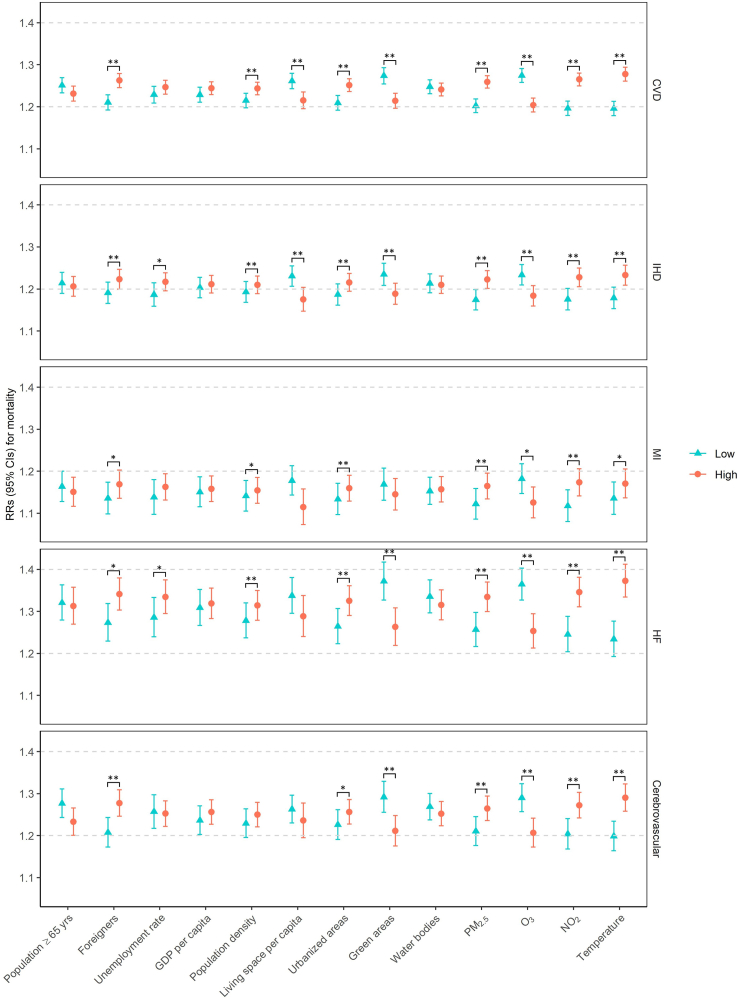
**Heat effects on cause-specific cardiovascular mortality at low and high levels of effect modifiers (25th and 75th percentile of the modifier's distribution) from single-predictor meta-regressions**. Heat effects at low and high levels of effect modifiers are represented as relative risks estimated from single-predictor meta-regression with districts as the random effect. *∗p*-value of Wald test <0.05; ∗∗*p*-value of Wald test <0.01. CI, confidence interval; CVD, cardiovascular disease; GDP, gross domestic product; HF, heart failure; IHD, ischemic heart disease; MI, myocardial infarction; NO_2_, nitrogen dioxide; O_3_, ozone; PM_2.5_, particulate matter with a diameter of 2.5 μm or less; RR, relative risk.

**Fig. 2:**
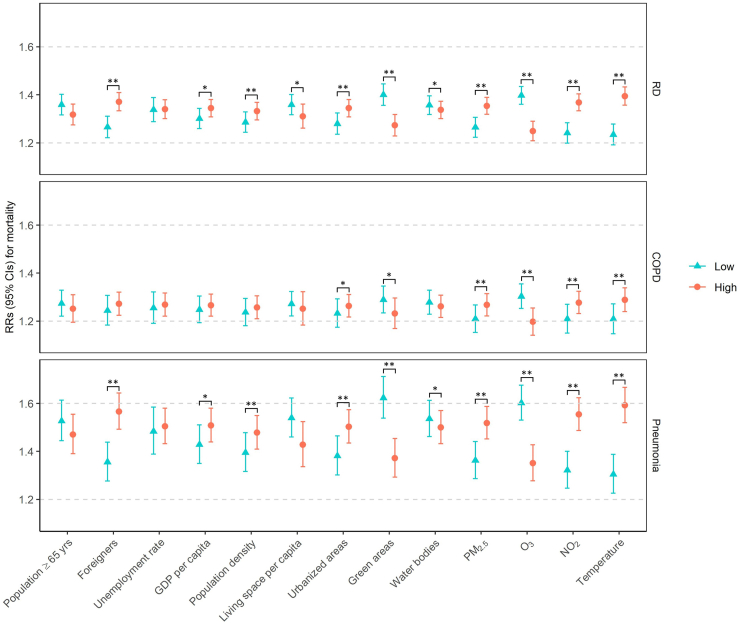
**Heat effects on cause-specific respiratory mortality at low and high levels of effect modifiers (25th and 75th percentile of the modifier's distribution) from single-predictor meta-regressions**. Heat effects at low and high levels of effect modifiers are represented as relative risks estimated from single-predictor meta-regression with districts as the random effect. *∗p*-value of Wald test <0.05; ∗∗*p*-value of Wald test <0.01. CI, confidence interval; COPD, chronic obstructive pulmonary disease; GDP, gross domestic product; NO_2_, nitrogen dioxide; O_3_, ozone; PM_2.5_, particulate matter with a diameter of 2.5 μm or less; RD, respiratory disease; RR, relative risk.

In single-predictor meta-regressions, we observed the most significant heat effect modification by the degree of urbanization, long-term exposure levels of air pollution, and mean temperature over the study period (whole-year temperature). Specifically, the heat-related mortality risks for all examined causes were stronger in districts with higher percentages of urbanized areas, higher levels of PM_2.5_ and NO_2_, higher mean temperature, and lower levels of O_3_ (Figs. 1 and 2, Supplementary Tables S7–S14). For example, heat exposure was associated with RRs of 1.21 (95% CI: 1.19, 1.23) and 1.25 (95% CI: 1.24, 1.27) for CVD mortality in districts with low (4.9%) and high (15.8%) percentages of urbanized areas, respectively. In addition, factors related to urbanization, including high proportions of foreigners, high population density, and less green areas, characterized districts with stronger heat effects on most causes of mortality. Among the examined endpoints, the differences in heat effect estimates between districts with low vs. high levels of characteristics were most notable for CVD, HF, RD, and pneumonia mortality. In contrast, they were less evident for MI and COPD mortality (Figs. 1 and 2).

The heat effect modifications by long-term air pollution and temperature exposures remained significant in the two-predictor meta-regressions with adjustment for the percentage of urbanized areas (Supplementary Tables S7–S14). Stable modifications were also observed for the proportion of foreigners and the amount of green areas per 100,000 persons on the associations of heat with mortality from cerebrovascular diseases, RD, and pneumonia. We did not find significant effect modifications by other characteristics after adjusting for urbanization.

**Fig. 3:**
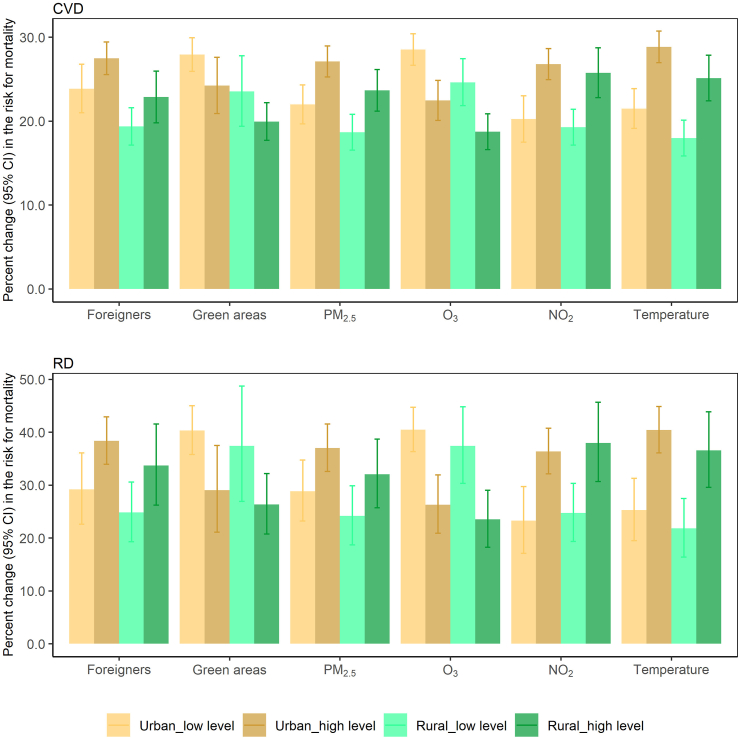
**Heat effects on cardiovascular (top) and respiratory (bottom) mortality at low and high levels of effect modifiers (25th and 75th percentile of the modifier's distribution) in urban and rural areas**. Heat effects at low and high levels of effect modifiers are represented as percent changes in mortality risk estimated from meta-regression incorporating type of district and the investigated characteristic. Characteristics showing significant effect modification in both single- and two-predictor meta-regressions were selected. CI, confidence interval; CVD, cardiovascular disease; PM_2.5_, particulate matter with a diameter of 2.5 μm or less; RD, respiratory disease.

Modifications of the heat effects on CVD and RD mortality by the proportion of foreigners, green areas, air pollution, and temperature, which were significant modifiers in both single- and two-predictor meta-regression for at least one death cause, were similar between urban and rural districts (Fig. 3).

### Sensitivity analysis

The heat effects on cause-specific mortality became stronger when restricting the analyses to the three hottest months from June to August, extending the lag time to three days, or using the MMT instead of the 75th percentile for effect estimation (Supplementary Figure S2). After adjusting for air pollution, we observed reduced heat effects except for IHD, MI, and COPD mortality.

## Discussion

Our study comprehensively assessed heat effects on cause-specific cardiopulmonary mortality and related vulnerability factors in Germany, using nationwide data aggregated at the district level. Our results revealed associations between heat exposure and increased mortality risks for all examined causes during the warm season. The heat impact was overall stronger for RD than CVD mortality. Regarding specific sub-causes of cardiopulmonary mortality, the most pronounced heat effects were found for HF and pneumonia mortality. Greater susceptibility to heat was observed among individuals aged ≥75 years and females, as well as in districts with higher degrees of urbanization, elevated levels of PM_2.5_ or NO_2_, and higher mean air temperatures over the study period. Notable disparities in heat vulnerability factors were found across various death causes. Moreover, the modification of heat effects on CVD and RD mortality by district-level characteristics remained consistent in urban and rural districts.

Our research demonstrated increased cardiopulmonary mortality risks associated with heat exposure, with a stronger impact on RD than CVD mortality. These findings were consistent with previous studies conducted in major cities.^19^^,^^20^ For instance, in a multi-country analysis involving 482 cities, the overall heat effect estimates showed a 6.4% (95% CI: 6.3%, 6.4%) increase in CVD mortality and an 8.4% (95% CI: 8.4%, 8.5%) increase in RD mortality for an increment in the 2-day average temperature from the 75th to the 99th percentile.^15^ The German-specific estimates derived from 14 cities in this study were 24.1% (95% CI: 24.0%, 24.2%) for CVD and 32.3% (95% CI: 32.1%, 32.5%) for RD mortality, thus comparable with our results. The underlying mechanisms for the stronger heat impact on RD mortality remain unclear. One potential explanation could be the rapid deterioration in health conditions among patients with chronic respiratory diseases under heat stress. It has been suggested that COPD patients are at a higher risk of developing pulmonary vascular resistance due to peripheral pooling of blood or hypovolemia during heat exposure.^21^

We observed the strongest heat effect on HF mortality compared to other subtypes of CVD, suggesting that HF patients were particularly vulnerable to heat stress. Similar results were reported in another German study conducted in three southern cities, where heat was most significantly associated with HF mortality but not with MI mortality.^22^ An analysis of CVD mortality data from 567 cities in 27 countries also found that extreme high temperatures imposed the greatest burden on HF mortality.^23^ For every 1000 HF deaths, 2.6 (95% eCI: 2.4, 2.8) were attributed to extremely high temperatures above the 97.5% percentile. Chronic heart failure (CHF) has been identified as a strong risk indicator of in-hospital heat-related mortality.^24^ The heat intolerance in patients with CHF could be due to their hemodynamic derangements and cardiac reserve limitations. Consequently, the increase in cardiac output during heat exposure might be insufficient for effectively perfusing the skin to dissipate heat while maintaining adequate perfusion pressure and blood flow to vital organs.^25^ In addition, CHF patients have demonstrated reduced cutaneous vasodilator responses to high temperatures independent of cardiac output.^25^ Quantifying the heat impacts on cause-specific CVD mortality has important implications for understanding the physiological mechanisms underlying the heat-mortality association. Our study observed a notably higher estimate for HF mortality than other CVD sub-causes. It can therefore be speculated that heat-related blood flow redirection and consequential increased cardiac workload might be a major pathway for CHF patients. This finding also highlighted the need to adjust medications prescribed to CHF patients, such as diuretics, during extreme heat exposure to prevent dehydration and electrolyte imbalance.^26^

Pneumonia, as a lower respiratory tract infection, has been considered a climate-sensitive disease and exhibits seasonal variations. A meta-analysis of temperature impacts among older adults showed the greatest risk associated with cold-induced pneumonia morbidity.^11^ However, evidence for the effect of heat on pneumonia mortality is still limited. In line with our results, a study in 16 Chinese cities found a heat-related increase in pneumonia mortality, which was higher than the estimates for COPD and asthma mortality.^27^ In a time-series analysis involving 12 U.S. cities, the adverse effect of high temperatures on pneumonia mortality was only detected in cold cities but not in hot cities.^28^ High air temperature and relative humidity on hot days favor the survival and propagation of bacteria and viruses, elevating the risk of infection in susceptible individuals.^29^ Furthermore, pre-existing health conditions associated with higher susceptibility to heat stress, including CVD, COPD, neurologic disease, and mobility impairment, have been identified as major prognostic factors of mortality in pneumonia patients.^30^^,^^31^ Our result of the strong heat impact on pneumonia mortality highlights the interplay between pathogens, weather, and comorbidities. Further, it implies the necessity to implement multifaceted measures to reduce the climate-related health burden.

In our study, the heat effects on cardiopulmonary mortality were stronger in the older age group and females, as reported in previous investigations.^3^^,^^7^^,^^9^^,^^27^ The greater heat vulnerability of older people can be attributed to age-related declines in thermoregulation capacity. Research has demonstrated that older people exhibit diminished heat sensitivity, reduced skin blood flow, and lower sweat rates during heat exposure.^32^ These dysfunctions can be exacerbated by medications that interfere with normal thermoregulation, such as diuretics, commonly prescribed to manage hypertension and HF.^33^ Besides, a higher prevalence of chronic health conditions, including cardiopulmonary diseases, diabetes, and neurodegenerative diseases, can amplify the risk of heat-related mortality among older people. Our sex-stratified analyses within the same age group revealed a consistent pattern of stronger heat effects among the females, suggesting a greater susceptibility to heat that was independent of the potential older ages at death in the females. The sex difference may be related to the lower heat dissipation capacity in women because they tend to have a higher percentage of body fat and a lower sweat rate.^34^ Besides, menstrual cycle hormones can lead to higher body temperatures and influence thermoregulation. During menopause, the decline in estrogen levels may impact skin blood flow control, exacerbating heat-related impacts for older women.^35^

Our finding of the greater heat vulnerability in highly urbanized districts could be explained by the urban heat island (UHI) effect. Urban areas usually have higher coverages of impervious surfaces, which absorb and retain heat more effectively than natural landscapes during the daytime and release the heat slowly at night. This leads to microclimates in cities that are, on average, warmer than their surrounding rural areas, especially at nighttime. Anthropogenic heat release, such as energy consumption, vehicular emissions, and industrial processes, exacerbate the UHI effect. As a result, urban residents are exposed to prolonged periods of extreme heat, increasing the risk of heat-related illnesses and mortality.

Our study identified air pollution and greenness as two modifiable environmental factors that can potentially mitigate the health impacts of heat. Long-term exposure to air pollution has been associated with exacerbating cardiovascular conditions and impaired respiratory function,^36^^,^^37^ making vulnerable populations more susceptible to heat-related cardiopulmonary deaths. While the average concentrations of PM_2.5_ and NO_2_ in our study area were below the annual limits of the European Union air quality standards, the PM_2.5_ levels in all German districts were above the 2021 World Health Organization (WHO) reference limit of 5 μg/m^3^; the majority of districts also exceeded the 10 μg/m^3^ limit for annual NO_2_ concentrations. Implementing stringent air quality regulations according to WHO air quality guidelines can not only reduce the adverse health effects of air pollution but also enhance public health resilience to extreme heat. In contrast to PM_2.5_ and NO_2_, we observed stronger heat effects on cardiopulmonary mortality in districts with low O_3_ levels. This could be explained by the local depletion of O_3_ by high levels of NO_2_ from traffic emissions, leading to lower O_3_ concentrations in these districts.^38^ Therefore, the effect modification by O_3_ in our study might be due to its negative correlation with NO_2_ (Spearman correlation coefficient = −0.77), rather than a protective effect of high O_3_ levels.

Greenness has been suggested to mitigate the heat impacts by cooling down the microclimate through shading surfaces and evapotranspiration. A European study involving 93 cities estimated that increasing city tree coverage to 30% (a mean increase of 17.7% in tree coverage) could reduce summer temperature by 0.4 °C and prevent 2644 (95% CI: 2444–2824) premature deaths in 2015.^39^ Besides, higher exposure to greenness has been shown to promote mental health, social engagement, and physical activity, which can lower the mortality risk.^40^

One strength of our study is the use of German-wide data with full coverage of urban and rural districts, thereby enhancing the generalizability of our findings compared to previous studies confined to major cities. Our comprehensive evaluation of heat effects on cause-specific cardiopulmonary mortality and respective heat vulnerability factors may inform healthcare providers of the patients most at risk on hot days and facilitate the development of targeted interventions. Additionally, our application of high-resolution temperature data for exposure assessment minimized measurement errors and the related bias in the effect estimates.

Our study also has a few limitations. First, our analysis was conducted exclusively in Germany, potentially limiting the applicability of our results to regions with different climatic and sociodemographic conditions. Second, our meta-regressions used averaged district-specific characteristics over the study period and did not account for potential temporal variations in heat vulnerability factors. Third, we did not adjust for the potential confounding effect of seasonal allergies and pollen counts in the quasi-Poisson regression analysis due to data unavailability. Given the association of heat with longer pollen seasons and higher allergenic pollen concentrations, which may synergistically exacerbate respiratory conditions such as asthma and COPD,^41^^,^^42^ this lack of adjustment might lead to an overestimation of the heat effects on respiratory mortality. Fourth, based on aggregated data, our study provides insights at the population level and does not support individual-level inferences. Fifth, we did not explore other cardiopulmonary disease subtypes, such as asthma, due to a limited number of deaths, which necessitates further investigation.

### Conclusions

This nationwide study reveals increased risks of cause-specific cardiopulmonary mortality associated with heat in Germany. Notably, advanced age, female sex, high degrees of urbanization, and high levels of air pollution and temperature emerged as major heat vulnerability factors. These factors exhibited variability across different cardiopulmonary disease subtypes while remaining generally consistent between urban and rural regions. Our findings provided insights for evidence-based disease management and policy development to reduce the burden of heat-related diseases in the context of climate change.

## Contributors

**Siqi Zhang**: Conceptualization, Methodology, Software, Formal analysis, Investigation, Writing-Original Draft, Writing-Review & Editing, Visualization. **Susanne Breitner**: Conceptualization, Methodology, Resources, Data Curation, Writing-Review & Editing. **Francesca de’ Donato**: Conceptualization, Methodology, Writing-Review & Editing. **Massimo Stafoggia:** Conceptualization, Methodology, Writing-Review & Editing. **Nikolaos Nikolaou**: Resources, Data Curation, Writing-Review & Editing. **Kristin Aunan:** Conceptualization, Writing-Review & Editing, Project administration, Funding acquisition. **Annette Peters**: Conceptualization, Writing-Review & Editing. **Alexandra Schneider**: Conceptualization, Methodology, Resources, Data Curation, Writing-Review & Editing, Project administration, Funding acquisition, Supervision.

## Data sharing statement

Mortality data can be obtained from the Research Data Centre of the Federal Statistical Office and Statistical Offices of the Federal States (https://www.forschungsdatenzentrum.de/) upon request. Meteorological data are available from the corresponding author upon reasonable request. District-level characteristic data are publicly available from the INKAR database (https://www.inkar.de) of the Federal Office for Building and Regional Planning (BBSR) and the Regional Atlas database (https://regionalatlas.statistikportal.de).

## Declaration of interests

The authors declare no conflicts of interest.
