# Supplementary material for: Heat and cause-specific cardiopulmonary mortality in Germany: a case-crossover study using small-area assessment
Source: Lancet Reg Health Eur. 2024 Sep 6;46:101049. doi: 10.1016/j.lanepe.2024.101049 (PMC11406445; doi:10.1016/j.lanepe.2024.101049)
Supplement: Supplementary material [file mmc1.docx]

**SUPPLEMENTARY MATERIAL**

**Table of contents**

[Table S1. Number and percentage^a^ of cardiovascular and respiratory deaths by age group and sex from May to September of the study period (2000-2016). 2](#_Toc168972022)

[Table S2. Distribution and Spearman correlation coefficients of daily mean air temperature and air pollutant concentrations during the warm season (May to September) of 2000 to 2016. 3](#_Toc168972023)

[Table S3. Spearman correlation coefficients of district-level characteristics. 4](#_Toc168972024)

[Table S4. Description (Mean ± SD) of district characteristics in urban and rural areas. 5](#_Toc168972025)

[Table S5. *P*-value of the two-sided Z-test for statistical significance of the between-group difference in heat effect estimates by age and sex. 6](#_Toc168972026)

[Table S6. Pooled relative risks (95% confidence intervals) of cause-specific cardiopulmonary mortality for an increase in air temperature from the 75th to the 99th percentile in different combinations of age and sex subgroups. 7](#_Toc168972027)

[Table S7. Heat effects on cardiovascular mortality at low and high levels of effect modifiers (25th and 75th percentile of the modifier’s distribution) from single-predictor (left) and two-predictor (right, with adjustment for degree of urbanization) meta-regressions. 8](#_Toc168972028)

[Table S8. Heat effects on ischemic heart disease mortality at low and high levels of effect modifiers (25th and 75th percentile of the modifier’s distribution) from single-predictor (left) and two-predictor (right, with adjustment for degree of urbanization) meta-regressions. 9](#_Toc168972029)

[Table S9. Heat effects on myocardial infarction mortality at low and high levels of effect modifiers (25th and 75th percentile of the modifier’s distribution) from single-predictor (left) and two-predictor (right, with adjustment for degree of urbanization) meta-regressions. 10](#_Toc168972030)

[Table S10. Heat effects on heart failure mortality at low and high levels of effect modifiers (25th and 75th percentile of the modifier’s distribution) from single-predictor (left) and two-predictor (right, with adjustment for degree of urbanization) meta-regressions. 11](#_Toc168972031)

[Table S11. Heat effects on cerebrovascular mortality at low and high levels of effect modifiers (25th and 75th percentile of the modifier’s distribution) from single-predictor (left) and two-predictor (right, with adjustment for degree of urbanization) meta-regressions. 12](#_Toc168972032)

[Table S12. Heat effects on respiratory mortality at low and high levels of effect modifiers (25th and 75th percentile of the modifier’s distribution) from single-predictor (left) and two-predictor (right, with adjustment for degree of urbanization) meta-regressions. 13](#_Toc168972033)

[Table S13. Heat effects on COPD mortality at low and high levels of effect modifiers (25th and 75th percentile of the modifier’s distribution) from single-predictor (left) and two-predictor (right, with adjustment for degree of urbanization) meta-regressions. 14](#_Toc168972034)

[Table S14. Heat effects on pneumonia mortality at low and high levels of effect modifiers (25th and 75th percentile of the modifier’s distribution) from single-predictor (left) and two-predictor (right, with adjustment for degree of urbanization) meta-regressions. 15](#_Toc168972035)

[Figure S1. Cumulative exposure-response relationships between air temperature and cause-specific cardiopulmonary mortality across Germany. 16](#_Toc168972036)

[Figure S2. Pooled relative risk (95% CI) of cause-specific cardiovascular and respiratory mortality for an increase in air temperature from the 75^th^ to the 99^th^ percentile in sensitivity analyses. 17](#_Toc168972037)

# Table S1. Number and percentage^a^ of cardiovascular and respiratory deaths by age group and sex from May to September of the study period (2000-2016).

| **Outcome** | **Age ≥ 65 years** | **Age ≥ 75 years** | **Male** | **Female** |
| --- | --- | --- | --- | --- |
| **CVD** | 1,891,161 (92∙2) | 1,616,927 (78∙8) | 836,034 (40∙8) | 1,214,730 (59∙2) |
| IHD | 717,188 (90∙1) | 587,615 (73∙8) | 383,314 (48∙1) | 413,046 (51∙9) |
| MI | 264,916 (83∙9) | 198,754 (63∙0) | 169,411 (53∙7) | 146,218 (46∙3) |
| HF | 279,444 (96∙2) | 256,089 (88∙1) | 93,007 (32∙0) | 197,546 (68∙0) |
| Cerebrovascular | 340,918 (94∙5) | 295,572 (81∙9) | 131,461 (36∙4) | 229,403 (63∙6) |
| **RD** | 272,486 (91∙1) | 218,984 (73∙2) | 153,746 (51∙4) | 145,503 (48∙6) |
| COPD | 122,758 (89∙4) | 90,373 (65∙8) | 80,336 (58∙5) | 56,915 (41∙5) |
| Pneumonia | 93,269 (94∙1) | 81,734 (82∙5) | 45,380 (45∙8) | 53,751 (54∙2) |

^a^Percentage of deaths in each subgroup relative to the cause-specific total number of deaths.

COPD=chronic obstructive pulmonary disease, CVD=cardiovascular disease, HF=heart failure, IHD=ischemic heart disease, MI=myocardial infarction, RD=respiratory disease.

# Table S2. Distribution and Spearman correlation coefficients of daily mean air temperature and air pollutant concentrations^a^ during the warm season (May to September) of 2000 to 2016.

| **Exposure** | **Mean ± SD** | **Min.** | **Median** | **P75** | **P99** | **Max.** | **Spearman correlation** | | |
| --- | --- | --- | --- | --- | --- | --- | --- | --- | --- |
|  |  |  |  |  |  |  | **Temp** | **PM_2∙5_** | **O_3_** |
| Temperature (°C) | 15∙7 ± 3∙9 | 0∙5 | 15∙7 | 18∙3 | 24∙8 | 31∙0 |  |  |  |
| PM_2∙5_ (µg/m^3^) | 9∙3 ± 4∙4 | 0∙5 | 8∙4 | 11∙5 | 23∙7 | 59∙6 | -0∙26 |  |  |
| O_3_ (µg/m^3^) | 59∙2 ± 17∙4 | 1∙7 | 58∙3 | 69∙6 | 106∙0 | 155∙2 | 0∙52 | -0∙36 |  |
| NO_2_ (µg/m^3^) | 10∙3 ± 6∙7 | 0∙6 | 8∙5 | 12∙9 | 34∙1 | 80∙7 | -0∙44 | 0∙52 | -0∙67 |

^a^Distribution of air pollutant concentrations and Spearman correlation coefficients were based on data for the years 2004-2016 due to air pollution data availability.

NO_2_=nitrogen dioxide, O_3_=ozone, PM_2∙5_=particulate matter with a diameter of 2∙5 µm or less, SD=standard deviation, Temp=temperature.

# Table S3. Spearman correlation coefficients of district-level characteristics.

| **Characteristic** | **Age≥65** | **Foreigners** | **Unemployment** | **GDP** | **Population density** | **Living space** | **Urbanized areas** | **Green areas** | **Water bodies** | **PM_2∙5_** | **O_3_** | **NO_2_** |
| --- | --- | --- | --- | --- | --- | --- | --- | --- | --- | --- | --- | --- |
| Foreigners | -0∙34 |  |  |  |  |  |  |  |  |  |  |  |
| Unemployment | 0∙56 | -0∙23 |  |  |  |  |  |  |  |  |  |  |
| GDP | -0∙18 | 0∙66 | -0∙15 |  |  |  |  |  |  |  |  |  |
| Population density | -0∙08 | 0∙72 | 0∙22 | 0∙55 |  |  |  |  |  |  |  |  |
| Living space | 0∙04 | -0∙31 | -0∙49 | -0∙25 | -0∙53 |  |  |  |  |  |  |  |
| Urbanized areas | 0∙00 | 0∙65 | 0∙24 | 0∙51 | 0∙94 | -0∙48 |  |  |  |  |  |  |
| Green areas | 0∙14 | -0∙64 | 0∙11 | -0∙48 | -0∙60 | 0∙35 | -0∙67 |  |  |  |  |  |
| Water bodies | 0∙13 | -0∙28 | 0∙29 | -0∙18 | -0∙21 | -0∙08 | -0∙24 | 0∙46 |  |  |  |  |
| PM_2∙5_^a^ | -0∙04 | 0∙44 | 0∙33 | 0∙31 | 0∙71 | -0∙51 | 0∙71 | -0∙44 | -0∙15 |  |  |  |
| O_3_^a^ | 0∙25 | -0∙48 | 0∙05 | -0∙36 | -0∙53 | 0∙25 | -0∙55 | 0∙50 | 0∙27 | -0∙68 |  |  |
| NO_2_^a^ | -0∙16 | 0∙75 | 0∙00 | 0∙51 | 0∙77 | -0∙36 | 0∙76 | -0∙69 | -0∙32 | 0∙71 | -0∙77 |  |
| Temperature^b^ | -0∙09 | 0∙31 | 0∙31 | 0∙20 | 0∙49 | -0∙27 | 0∙51 | -0∙36 | -0∙03 | 0∙66 | -0∙53 | 0∙54 |

^a^Long-term air pollutant averages were calculated based on the whole-year daily mean air pollutant concentrations from 2004 to 2016.

^b^Mean temperature during the study period was calculated based on the whole-year daily mean temperatures from 2000 to 2016.

GDP=gross domestic product, NO_2_=nitrogen dioxide, O_3_=ozone, PM_2∙5_=particulate matter with a diameter of 2∙5 µm or less.

# Table S4. Description (Mean ± SD) of district characteristics in urban and rural areas.

| **Characteristic** | **Urban areas** | **Rural areas** | ***p-value*^a^** |
| --- | --- | --- | --- |
| Population aged≥65 years (%) | 19∙6 ± 1∙7 | 20∙4 ± 2∙2 | < 0∙001 |
| Foreigners (%) | 10∙1 ± 4∙6 | 5∙1 ± 2∙7 | < 0∙001 |
| Unemployment rate (%) | 7∙6 ± 3∙1 | 8∙3 ± 4∙1 | 0∙47 |
| GDP per capita (€1,000) | 32∙7 ± 14∙6 | 25∙4 ± 8∙9 | < 0∙001 |
| Population density (persons/km^2^) | 825 ± 794 | 214 ± 264 | < 0∙001 |
| Living space per capita (m^2^) | 43∙3 ± 3∙6 | 45∙4 ± 3∙6 | < 0∙001 |
| Urbanized area (%) | 16∙7 ± 11∙6 | 6∙7 ± 6 | < 0∙001 |
| Green areas (km^2^/100,000 persons) | 232∙4 ± 193∙4 | 834∙5 ± 469∙4 | < 0∙001 |
| Water bodies (km^2^/100,000 persons) | 2∙1 ± 4∙2 | 9∙4 ± 16∙5 | < 0∙001 |
| PM_2∙5_ (μg/m^3^) | 12∙9 ± 1∙8 | 11∙7 ± 1∙1 | < 0∙001 |
| O_3_ (μg/m^3^) | 45∙3 ± 5∙3 | 49∙6 ± 4∙5 | < 0∙001 |
| NO_2_ (μg/m^3^) | 18∙4 ± 5∙7 | 11∙8 ± 2∙7 | < 0∙001 |
| Temperature (°C) | 9∙7 ± 0∙8 | 9∙1 ± 0∙7 | < 0∙001 |

^a^*p*-values for the differences between urban and rural areas were derived using Kruskal-Wallis rank sum tests

GDP=gross domestic product, NO_2_=nitrogen dioxide, O_3_=ozone, PM_2∙5_=particulate matter with a diameter of 2∙5 µm or less, SD=standard deviation.

# Table S5. *P*-value of the two-sided Z-test for statistical significance of the between-group difference in heat effect estimates by age and sex.

| **Mortality** | **Age ≥ 65y vs. entire population** | **Age ≥ 75y vs. entire population** | **Males vs. females** |
| --- | --- | --- | --- |
| **CVD** | 0∙53 | 0∙13 | 0∙01 |
| IHD | 0∙49 | 0∙17 | 0∙01 |
| MI | 0∙65 | 0∙17 | 0∙002 |
| HF | 1∙00 | 0∙70 | 0∙60 |
| Cerebrovascular | 1∙00 | 0∙67 | 0∙13 |
| **RD** | 0∙72 | 0∙16 | 0∙05 |
| COPD | 0∙76 | 0∙46 | 0∙21 |
| Pneumonia | 0∙70 | 0∙33 | 0∙14 |

COPD=chronic obstructive pulmonary disease, CVD=cardiovascular disease, HF=heart failure, IHD=ischemic heart disease, MI=myocardial infarction, RD=respiratory disease.

# Table S6. Pooled relative risks (95% confidence intervals) of cause-specific cardiopulmonary mortality for an increase in air temperature from the 75th to the 99th percentile in different combinations of age and sex subgroups.

| **Mortality** | **Age ≥ 65y** | | |  | **Age ≥ 75y** | | |
| --- | --- | --- | --- | --- | --- | --- | --- |
|  | Males | Females | *p*-diff |  | Males | Females | *p*-diff |
| **CVD** | 1∙23 (1∙21, 1∙25) | 1∙26 (1∙24, 1∙28) | 0∙04 |  | 1∙25 (1∙22, 1∙27) | 1∙27 (1∙25, 1∙29) | 0∙28 |
| IHD | 1∙18 (1∙15, 1∙21) | 1∙25 (1∙22, 1∙28) | 0∙001 |  | 1∙20 (1∙16, 1∙23) | 1∙26 (1∙22, 1∙29) | 0∙04 |
| MI | 1∙13 (1∙08, 1∙17) | 1∙21 (1∙17, 1∙26) | 0∙02 |  | 1∙16 (1∙10, 1∙22) | 1∙23 (1∙18, 1∙28) | 0∙09 |
| HF | 1∙31 (1∙25, 1∙37) | 1∙33 (1∙29, 1∙37) | 0∙60 |  | 1∙34 (1∙27, 1∙41) | 1∙33 (1∙29, 1∙38) | 0∙81 |
| Cerebrovascular | 1∙29 (1∙24, 1∙34) | 1∙24 (1∙20, 1∙27) | 0∙13 |  | 1∙29 (1∙24, 1∙35) | 1∙24 (1∙21, 1∙28) | 0∙10 |
| **RD** | 1∙31 (1∙26, 1∙36) | 1∙39 (1∙33, 1∙45) | 0∙05 |  | 1∙34 (1∙28, 1∙39) | 1∙42 (1∙36, 1∙48) | 0∙07 |
| COPD | 1∙24 (1∙18, 1∙31) | 1∙31 (1∙23, 1∙39) | 0∙18 |  | 1∙26 (1∙19, 1∙35) | 1∙38 (1∙28, 1∙47) | 0∙06 |
| Pneumonia | 1∙47 (1∙36, 1∙59) | 1∙56 (1∙46, 1∙66) | 0∙25 |  | 1∙51 (1∙40, 1∙63) | 1∙58 (1∙48, 1∙68) | 0∙37 |

Conditional quasi-Poisson regression model with adjustment for a three-way interaction between the year, month, and day of the week was used to estimate the district-specific regression coefficients for a temperature increase from the 75th to the 99th percentile in each subgroup. Multivariate meta-analysis was then employed to derive the overall coefficient across districts. *p*-diff was the *p* value of the two-sided Z-test for statistical significance of the between-group difference in heat effect estimates by sex.

COPD=chronic obstructive pulmonary disease, CVD=cardiovascular disease, HF=heart failure, IHD=ischemic heart disease, MI=myocardial infarction, RD=respiratory disease.

# Table S7. Heat effects on cardiovascular mortality at low and high levels of effect modifiers (25th and 75th percentile of the modifier’s distribution) from single-predictor (left) and two-predictor (right, with adjustment for degree of urbanization) meta-regressions.

| **Characteristic** | **Single-predictor model** | | | | | |  | **Two-predictor model** | | |
| --- | --- | --- | --- | --- | --- | --- | --- | --- | --- | --- |
|  | **25th percentile** | |  | **75th percentile** | | ***p*-Wald** |  | **25th percentile** | **75th percentile** | ***p*-Wald** |
|  | **Value** | **RR (95% CI)** |  | **Value** | **RR (95% CI)** |  |  | **RR (95% CI)** | **RR (95% CI)** |  |
| Population aged≥65 years (%) | 18∙7 | 1∙25 (1∙23, 1∙27) |  | 21∙2 | 1∙23 (1∙21, 1∙25) | 0∙17 |  | 1∙23 (1∙21, 1∙25) | 1∙21 (1∙19, 1∙23) | 0∙29 |
| Foreigners (%) | 4∙4 | 1∙21 (1∙19, 1∙23) |  | 10∙5 | 1∙26 (1∙25, 1∙28) | <0∙001 |  | 1∙21 (1∙19, 1∙23) | 1∙23 (1∙21, 1∙26) | 0∙28 |
| Unemployment rate (%) | 5∙1 | 1∙23 (1∙21, 1∙25) |  | 9∙9 | 1∙25 (1∙23, 1∙26) | 0∙28 |  | 1∙22 (1∙20, 1∙24) | 1∙22 (1∙20, 1∙24) | 0∙75 |
| GDP per capita (€1,000) | 21∙6 | 1∙23 (1∙21, 1∙25) |  | 31∙5 | 1∙24 (1∙23, 1∙26) | 0∙06 |  | 1∙22 (1∙20, 1∙24) | 1∙22 (1∙20, 1∙23) | 0∙74 |
| Population density (persons/km^2^) | 123 | 1∙21 (1∙20, 1∙23) |  | 675 | 1∙24 (1∙23, 1∙26) | <0∙001 |  | -- | -- | -- |
| Living space per capita (m^2^) | 41∙2 | 1∙26 (1∙24, 1∙28) |  | 47∙0 | 1∙22 (1∙20, 1∙24) | 0∙001 |  | 1∙22 (1∙20, 1∙25) | 1∙21 (1∙19, 1∙23) | 0∙66 |
| Urbanized areas (%) | 4∙9 | 1∙21 (1∙19, 1∙23) |  | 15∙8 | 1∙25 (1∙24, 1∙27) | <0∙001 |  | -- | -- | -- |
| Green areas (km^2^/100,000 persons) | 119∙4 | 1∙27 (1∙25, 1∙29) |  | 781∙9 | 1∙21 (1∙20, 1∙23) | <0∙001 |  | 1∙24 (1∙21, 1∙26) | 1∙21 (1∙19, 1∙23) | 0∙16 |
| Water bodies (km^2^/100,000 persons) | 0∙4 | 1∙25 (1∙23, 1∙26) |  | 5∙8 | 1∙24 (1∙23, 1∙26) | 0∙05 |  | 1∙22 (1∙20,1∙24) | 1∙22 (1∙20, 1∙23) | 0∙36 |
| PM_2∙5_ (μg/m^3^) | 11∙4 | 1∙20 (1∙19, 1∙22) |  | 13∙1 | 1∙26 (1∙24, 1∙27) | <0∙001 |  | 1∙20 (1∙19, 1∙22) | 1∙26 (1∙24, 1∙28) | <0∙001 |
| O_3_ (μg/m^3^) | 43∙9 | 1∙27 (1∙26, 1∙29) |  | 51∙1 | 1∙20 (1∙19, 1∙22) | <0∙001 |  | 1∙26 (1∙23, 1∙28) | 1∙20 (1∙18, 1∙22) | <0∙001 |
| NO_2_ (μg/m^3^) | 11∙2 | 1∙20 (1∙18, 1∙21) |  | 18∙0 | 1∙26 (1∙25, 1∙28) | <0∙001 |  | 1∙20 (1∙18, 1∙21) | 1∙27 (1∙24, 1∙29) | <0∙001 |
| Temperature (°C) | 8∙9 | 1∙20 (1∙18, 1∙21) |  | 10∙0 | 1∙28 (1∙26, 1∙29) | <0∙001 |  | 1∙19 (1∙18, 1∙21) | 1∙26 (1∙24, 1∙28) | <0∙001 |

Heat effects at low and high levels of effect modifiers are represented as relative risks estimated from single- and two-predictor meta-regression with districts as the random effect.

CI=confidence interval, GDP=gross domestic product, NO_2_=nitrogen dioxide, O_3_=ozone, PM_2∙5_=particulate matter with a diameter of 2∙5 µm or less, RR=relative risk.

# Table S8. Heat effects on ischemic heart disease mortality at low and high levels of effect modifiers (25th and 75th percentile of the modifier’s distribution) from single-predictor (left) and two-predictor (right, with adjustment for degree of urbanization) meta-regressions.

| **Characteristic** | **Single-predictor model** | | | | | |  | **Two-predictor model** | | |
| --- | --- | --- | --- | --- | --- | --- | --- | --- | --- | --- |
|  | **25th percentile** | |  | **95^th^ percentile** | | ***p*-Wald** |  | **25th percentile** | **95^th^ percentile** | ***p*-Wald** |
|  | **Value** | **RR (95% CI)** |  | **Value** | **RR (95% CI)** |  |  | **RR (95% CI)** | **RR (95% CI)** |  |
| Population aged≥65 years (%) | 18∙7 | 1∙21 (1∙19, 1∙24) |  | 21∙2 | 1∙21 (1∙18, 1∙23) | 0∙88 |  | 1∙19 (1∙17, 1∙22) | 1∙19 (1∙16, 1∙22) | 0∙81 |
| Foreigners (%) | 4∙4 | 1∙19 (1∙17, 1∙22) |  | 10∙5 | 1∙22 (1∙20, 1∙25) | 0∙01 |  | 1∙19 (1∙16, 1∙21) | 1∙20 (1∙17, 1∙23) | 0∙72 |
| Unemployment rate (%) | 5∙1 | 1∙19 (1∙16, 1∙21) |  | 9∙9 | 1∙22 (1∙20, 1∙24) | 0∙05 |  | 1∙18 (1∙15, 1∙21) | 1∙20 (1∙17, 1∙23) | 0∙21 |
| GDP per capita (€1,000) | 21∙6 | 1∙20 (1∙18, 1∙23) |  | 31∙5 | 1∙21 (1∙19, 1∙23) | 0∙30 |  | 1∙20 (1∙17, 1∙22) | 1∙19 (1∙16, 1∙21) | 0∙65 |
| Population density (persons/km^2^) | 123 | 1∙19 (1∙17, 1∙22) |  | 675 | 1∙21 (1∙19, 1∙23) | <0∙001 |  | -- | -- | -- |
| Living space per capita (m^2^) | 41∙2 | 1∙23 (1∙21, 1∙26) |  | 47∙0 | 1∙18 (1∙15, 1∙20) | 0∙001 |  | 1∙21 (1∙18, 1∙25) | 1∙17 (1∙15, 1∙20) | 0∙20 |
| Urbanized areas (%) | 4∙9 | 1∙19 (1∙16, 1∙21) |  | 15∙8 | 1∙22 (1∙19, 1∙24) | <0∙001 |  | -- | -- | -- |
| Green areas (km^2^/100,000 persons) | 119∙4 | 1∙23 (1∙21, 1∙26) |  | 781∙9 | 1∙19 (1∙16, 1∙21) | <0∙001 |  | 1∙21 (1∙17, 1∙25) | 1∙19 (1∙16, 1∙21) | 0∙48 |
| Water bodies (km^2^/100,000 persons) | 0∙4 | 1∙21 (1∙19, 1∙24) |  | 5∙8 | 1∙21 (1∙19, 1∙23) | 0∙17 |  | 1∙19 (1∙17, 1∙22) | 1∙19 (1∙17, 1∙22) | 0∙73 |
| PM_2∙5_ (μg/m^3^) | 11∙4 | 1∙17 (1∙15, 1∙20) |  | 13∙1 | 1∙22 (1∙20, 1∙24) | <0∙001 |  | 1∙17 (1∙15, 1∙20) | 1∙24 (1∙21, 1∙27) | <0∙001 |
| O_3_ (μg/m^3^) | 43∙9 | 1∙23 (1∙21, 1∙26) |  | 51∙1 | 1∙18 (1∙16, 1∙21) | 0∙001 |  | 1∙22 (1∙19, 1∙25) | 1∙18 (1∙16, 1∙21) | 0∙08 |
| NO_2_ (μg/m^3^) | 11∙2 | 1∙18 (1∙15, 1∙20) |  | 18∙0 | 1∙23 (1∙21, 1∙25) | <0∙001 |  | 1∙17 (1∙15, 1∙20) | 1∙24 (1∙20, 1∙27) | 0∙02 |
| Temperature (°C) | 8∙9 | 1∙18 (1∙15, 1∙20) |  | 10∙0 | 1∙23 (1∙21, 1∙26) | <0∙001 |  | 1∙18 (1∙15, 1∙20) | 1∙22 (1∙19, 1∙25) | 0∙08 |

Heat effects at low and high levels of effect modifiers are represented as relative risks estimated from single- and two-predictor meta-regression with districts as the random effect.

CI=confidence interval, GDP=gross domestic product, NO_2_=nitrogen dioxide, O_3_=ozone, PM_2∙5_=particulate matter with a diameter of 2∙5 µm or less, RR=relative risk.

# Table S9. Heat effects on myocardial infarction mortality at low and high levels of effect modifiers (25th and 75th percentile of the modifier’s distribution) from single-predictor (left) and two-predictor (right, with adjustment for degree of urbanization) meta-regressions.

| **Characteristic** | **Single-predictor model** | | | | | |  | **Two-predictor model** | | |
| --- | --- | --- | --- | --- | --- | --- | --- | --- | --- | --- |
|  | **25th percentile** | |  | **75th percentile** | | ***p*-Wald** |  | **25th percentile** | **75th percentile** | ***p*-Wald** |
|  | **Value** | **RR (95% CI)** |  | **Value** | **RR (95% CI)** |  |  | **RR (95% CI)** | **RR (95% CI)** |  |
| Population aged≥65 years (%) | 18∙7 | 1∙16 (1∙13, 1∙20) |  | 21∙2 | 1∙15 (1∙12, 1∙19) | 0∙81 |  | 1∙14 (1∙10, 1∙19) | 1∙14 (1∙10, 1∙17) | 0∙97 |
| Foreigners (%) | 4∙4 | 1∙14 (1∙10, 1∙17) |  | 10∙5 | 1∙17 (1∙14, 1∙20) | 0∙02 |  | 1∙13 (1∙10, 1∙17) | 1∙15 (1∙10, 1∙20) | 0∙85 |
| Unemployment rate (%) | 5∙1 | 1∙14 (1∙10, 1∙18) |  | 9∙9 | 1∙16 (1∙13, 1∙19) | 0∙45 |  | 1∙13 (1∙09, 1∙17) | 1∙14 (1∙11, 1∙18) | 0∙29 |
| GDP per capita (€1,000) | 21∙6 | 1∙15 (1∙12, 1∙19) |  | 31∙5 | 1∙16 (1∙13, 1∙19) | 0∙17 |  | 1∙14 (1∙11, 1∙18) | 1∙13 (1∙10, 1∙17) | 0∙72 |
| Population density (persons/km^2^) | 123 | 1∙14 (1∙11, 1∙18) |  | 675 | 1∙15 (1∙12, 1∙19) | 0∙02 |  | -- | -- | -- |
| Living space per capita (m^2^) | 41∙2 | 1∙18 (1∙14, 1∙21) |  | 47∙0 | 1∙11 (1∙07, 1∙16) | 0∙06 |  | 1∙17 (1∙12, 1∙22) | 1∙11 (1∙07, 1∙16) | 0∙15 |
| Urbanized areas (%) | 4∙9 | 1∙13 (1∙10, 1∙17) |  | 15∙8 | 1∙16 (1∙13, 1∙19) | 0∙004 |  | -- | -- | -- |
| Green areas (km^2^/100,000 persons) | 119∙4 | 1∙17 (1∙13, 1∙21) |  | 781∙9 | 1∙14 (1∙11, 1∙18) | 0∙24 |  | 1∙13 (1∙07, 1∙19) | 1∙14 (1∙10, 1∙18) | 0∙49 |
| Water bodies (km^2^/100,000 persons) | 0∙4 | 1∙15 (1∙12, 1∙19) |  | 5∙8 | 1∙16 (1∙13, 1∙19) | 0∙22 |  | 1∙13 (1∙09, 1∙17) | 1∙14 (1∙10, 1∙17) | 0∙41 |
| PM_2∙5_ (μg/m^3^) | 11∙4 | 1∙12 (1∙09, 1∙16) |  | 13∙1 | 1∙16 (1∙13, 1∙20) | 0∙01 |  | 1∙12 (1∙09, 1∙16) | 1∙17 (1∙13, 1∙22) | 0∙03 |
| O_3_ (μg/m^3^) | 43∙9 | 1∙18 (1∙15, 1∙22) |  | 51∙1 | 1∙13 (1∙09, 1∙16) | 0∙02 |  | 1∙18 (1∙13, 1∙23) | 1∙12 (1∙09, 1∙16) | 0∙16 |
| NO_2_ (μg/m^3^) | 11∙2 | 1∙12 (1∙08, 1∙16) |  | 18∙0 | 1∙17 (1∙14, 1∙21) | <0∙001 |  | 1∙12 (1∙08, 1∙15) | 1∙19 (1∙14, 1∙25) | 0∙03 |
| Temperature (°C) | 8∙9 | 1∙14 (1∙10, 1∙17) |  | 10∙0 | 1∙17 (1∙14, 1∙21) | 0∙04 |  | 1∙13 (1∙09, 1∙17) | 1∙15 (1∙10, 1∙20) | 0∙26 |

Heat effects at low and high levels of effect modifiers are represented as relative risks estimated from single- and two-predictor meta-regression with districts as the random effect.

CI=confidence interval, GDP=gross domestic product, NO_2_=nitrogen dioxide, O_3_=ozone, PM_2∙5_=particulate matter with a diameter of 2∙5 µm or less, RR=relative risk.

# Table S10. Heat effects on heart failure mortality at low and high levels of effect modifiers (25th and 75th percentile of the modifier’s distribution) from single-predictor (left) and two-predictor (right, with adjustment for degree of urbanization) meta-regressions.

| **Characteristic** | **Single-predictor model** | | | | | |  | **Two-predictor model** | | |
| --- | --- | --- | --- | --- | --- | --- | --- | --- | --- | --- |
|  | **25th percentile** | |  | **75th percentile** | | ***p*-Wald** |  | **25th percentile** | **75th percentile** | ***p*-Wald** |
|  | **Value** | **RR (95% CI)** |  | **Value** | **RR (95% CI)** |  |  | **RR (95% CI)** | **RR (95% CI)** |  |
| Population aged≥65 years (%) | 18∙7 | 1∙32 (1∙28, 1∙36) |  | 21∙2 | 1∙31 (1∙27, 1∙36) | 0∙96 |  | 1∙28 (1∙23, 1∙32) | 1∙28 (1∙23, 1∙32) | 0∙95 |
| Foreigners (%) | 4∙4 | 1∙27 (1∙23, 1∙32) |  | 10∙5 | 1∙34 (1∙30, 1∙38) | 0∙02 |  | 1∙27 (1∙23, 1∙32) | 1∙28 (1∙23, 1∙34) | 0∙89 |
| Unemployment rate (%) | 5∙1 | 1∙29 (1∙24, 1∙33) |  | 9∙9 | 1∙33 (1∙29, 1∙37) | 0∙03 |  | 1∙27 (1∙22, 1∙32) | 1∙28 (1∙24, 1∙33) | 0∙33 |
| GDP per capita (€1,000) | 21∙6 | 1∙31 (1∙27, 1∙35) |  | 31∙5 | 1∙32 (1∙28, 1∙36) | 0∙91 |  | 1∙29 (1∙25, 1∙33) | 1∙26 (1∙22, 1∙31) | 0∙29 |
| Population density (persons/km^2^) | 123 | 1∙28 (1∙24, 1∙32) |  | 675 | 1∙31 (1∙28, 1∙35) | 0∙002 |  | -- | -- | -- |
| Living space per capita (m^2^) | 41∙2 | 1∙34 (1∙30, 1∙38) |  | 47∙0 | 1∙29 (1∙24, 1∙34) | 0∙36 |  | 1∙26 (1∙20, 1∙31) | 1∙29 (1∙24, 1∙34) | 0∙52 |
| Urbanized areas (%) | 4∙9 | 1∙26 (1∙22, 1∙31) |  | 15∙8 | 1∙33 (1∙29, 1∙36) | <0∙001 |  | -- | -- | -- |
| Green areas (km^2^/100,000 persons) | 119∙4 | 1∙37 (1∙33, 1∙42) |  | 781∙9 | 1∙26 (1∙22, 1∙31) | 0∙002 |  | 1∙31 (1∙24, 1∙38) | 1∙26 (1∙22, 1∙31) | 0∙34 |
| Water bodies (km^2^/100,000 persons) | 0∙4 | 1∙34 (1∙30, 1∙37) |  | 5∙8 | 1∙31 (1∙28, 1∙35) | 0∙07 |  | 1∙29 (1∙25, 1∙33) | 1∙28 (1∙24, 1∙32) | 0∙26 |
| PM_2∙5_ (μg/m^3^) | 11∙4 | 1∙26 (1∙22, 1∙30) |  | 13∙1 | 1∙33 (1∙30, 1∙37) | <0∙001 |  | 1∙26 (1∙22, 1∙30) | 1∙33 (1∙28, 1∙38) | 0∙02 |
| O_3_ (μg/m^3^) | 43∙9 | 1∙36 (1∙33, 1∙40) |  | 51∙1 | 1∙25 (1∙21, 1∙29) | <0∙001 |  | 1∙33 (1∙28, 1∙38) | 1∙25 (1∙21, 1∙29) | <0∙001 |
| NO_2_ (μg/m^3^) | 11∙2 | 1∙25 (1∙20, 1∙29) |  | 18∙0 | 1∙35 (1∙31, 1∙38) | <0∙001 |  | 1∙25 (1∙20, 1∙29) | 1∙34 (1∙28, 1∙40) | <0∙001 |
| Temperature (°C) | 8∙9 | 1∙23 (1∙19, 1∙28) |  | 10∙0 | 1∙37 (1∙33, 1∙41) | <0∙001 |  | 1∙23 (1∙19, 1∙27) | 1∙35 (1∙30, 1∙41) | <0∙001 |

Heat effects at low and high levels of effect modifiers are represented as relative risks estimated from single- and two-predictor meta-regression with districts as the random effect.

CI=confidence interval, GDP=gross domestic product, NO_2_=nitrogen dioxide, O_3_=ozone, PM_2∙5_=particulate matter with a diameter of 2∙5 µm or less, RR=relative risk.

# Table S11. Heat effects on cerebrovascular mortality at low and high levels of effect modifiers (25th and 75th percentile of the modifier’s distribution) from single-predictor (left) and two-predictor (right, with adjustment for degree of urbanization) meta-regressions.

| **Characteristic** | **Single-predictor model** | | | | | |  | **Two-predictor model** | | |
| --- | --- | --- | --- | --- | --- | --- | --- | --- | --- | --- |
|  | **25th percentile** | |  | **75th percentile** | | ***p*-Wald** |  | **25th percentile** | **75th percentile** | ***p*-Wald** |
|  | **Value** | **RR (95% CI)** |  | **Value** | **RR (95% CI)** |  |  | **RR (95% CI)** | **RR (95% CI)** |  |
| Population aged≥65 years (%) | 18∙7 | 1∙28 (1∙24, 1∙31) |  | 21∙2 | 1∙23 (1∙20, 1∙27) | 0∙10 |  | 1∙25 (1∙22, 1∙29) | 1∙22 (1∙18, 1∙25) | 0∙22 |
| Foreigners (%) | 4∙4 | 1∙21 (1∙17, 1∙24) |  | 10∙5 | 1∙28 (1∙25, 1∙31) | <0∙001 |  | 1∙21 (1∙17, 1∙24) | 1∙29 (1∙24, 1∙33) | 0∙01 |
| Unemployment rate (%) | 5∙1 | 1∙26 (1∙22, 1∙30) |  | 9∙9 | 1∙25 (1∙22, 1∙28) | 0∙84 |  | 1∙24 (1∙20, 1∙28) | 1∙23 (1∙19, 1∙26) | 0∙66 |
| GDP per capita (€1,000) | 21∙6 | 1∙24 (1∙20, 1∙27) |  | 31∙5 | 1∙26 (1∙23, 1∙29) | 0∙12 |  | 1∙23 (1∙19, 1∙26) | 1∙23 (1∙20, 1∙27) | 0∙26 |
| Population density (persons/km^2^) | 123 | 1∙23 (1∙20, 1∙26) |  | 675 | 1∙25 (1∙22, 1∙28) | 0∙10 |  | -- | -- | -- |
| Living space per capita (m^2^) | 41∙2 | 1∙26 (1∙23, 1∙30) |  | 47∙0 | 1∙24 (1∙20, 1∙28) | 0∙58 |  | 1∙23 (1∙18, 1∙27) | 1∙24 (1∙19, 1∙28) | 0∙84 |
| Urbanized areas (%) | 4∙9 | 1∙23 (1∙19, 1∙26) |  | 15∙8 | 1∙26 (1∙23, 1∙29) | 0∙01 |  | -- | -- | -- |
| Green areas (km^2^/100,000 persons) | 119∙4 | 1∙29 (1∙26, 1∙33) |  | 781∙9 | 1∙21 (1∙18, 1∙25) | 0∙002 |  | 1∙29 (1∙23, 1∙35) | 1∙21 (1∙17, 1∙25) | 0∙01 |
| Water bodies (km^2^/100,000 persons) | 0∙4 | 1∙27 (1∙24, 1∙30) |  | 5∙8 | 1∙25 (1∙22, 1∙28) | 0∙09 |  | 1∙25 (1∙21, 1∙29) | 1∙24 (1∙20, 1∙27) | 0∙29 |
| PM_2∙5_ (μg/m^3^) | 11∙4 | 1∙21 (1∙18, 1∙25) |  | 13∙1 | 1∙26 (1∙24, 1∙29) | <0∙001 |  | 1∙21 (1∙18, 1∙25) | 1∙28 (1∙24, 1∙33) | <0∙001 |
| O_3_ (μg/m^3^) | 43∙9 | 1∙29 (1∙26, 1∙32) |  | 51∙1 | 1∙21 (1∙17, 1∙24) | <0∙001 |  | 1∙29 (1∙24, 1∙33) | 1∙21 (1∙17, 1∙24) | 0∙004 |
| NO_2_ (μg/m^3^) | 11∙2 | 1∙20 (1∙17, 1∙24) |  | 18∙0 | 1∙27 (1∙24, 1∙30) | <0∙001 |  | 1∙20 (1∙17, 1∙24) | 1∙29 (1∙25, 1∙34) | 0∙004 |
| Temperature (°C) | 8∙9 | 1∙20 (1∙16, 1∙23) |  | 10∙0 | 1∙29 (1∙26, 1∙32) | <0∙001 |  | 1∙20 (1∙16, 1∙23) | 1∙29 (1∙25, 1∙34) | <0∙001 |

Heat effects at low and high levels of effect modifiers are represented as relative risks estimated from single- and two-predictor meta-regression with districts as the random effect.

CI=confidence interval, GDP=gross domestic product, NO_2_=nitrogen dioxide, O_3_=ozone, PM_2∙5_=particulate matter with a diameter of 2∙5 µm or less, RR=relative risk.

# Table S12. Heat effects on respiratory mortality at low and high levels of effect modifiers (25th and 75th percentile of the modifier’s distribution) from single-predictor (left) and two-predictor (right, with adjustment for degree of urbanization) meta-regressions.

| **Characteristic** | **Single-predictor model** | | | | | |  | **Two-predictor model** | | |
| --- | --- | --- | --- | --- | --- | --- | --- | --- | --- | --- |
|  | **25th percentile** | |  | **75th percentile** | | ***p*-Wald** |  | **25th percentile** | **75th percentile** | ***p*-Wald** |
|  | **Value** | **RR (95% CI)** |  | **Value** | **RR (95% CI)** |  |  | **RR (95% CI)** | **RR (95% CI)** |  |
| Population aged≥65 years (%) | 18∙7 | 1∙36 (1∙32, 1∙40) |  | 21∙2 | 1∙32 (1∙27, 1∙36) | 0∙27 |  | 1∙31 (1∙26, 1∙36) | 1∙28 (1∙23, 1∙32) | 0∙40 |
| Foreigners (%) | 4∙4 | 1∙27 (1∙22, 1∙31) |  | 10∙5 | 1∙37 (1∙33, 1∙41) | <0∙001 |  | 1∙26 (1∙22, 1∙31) | 1∙34 (1∙29, 1∙40) | 0∙06 |
| Unemployment rate (%) | 5∙1 | 1∙34 (1∙29, 1∙39) |  | 9∙9 | 1∙34 (1∙30, 1∙38) | 0∙23 |  | 1∙31 (1∙26, 1∙36) | 1∙28 (1∙23, 1∙32) | 0∙16 |
| GDP per capita (€1,000) | 21∙6 | 1∙30 (1∙26, 1∙34) |  | 31∙5 | 1∙34 (1∙31, 1∙38) | 0∙02 |  | 1∙28 (1∙24, 1∙33) | 1∙30 (1∙26, 1∙35) | 0∙52 |
| Population density (persons/km^2^) | 123 | 1∙29 (1∙24, 1∙33) |  | 675 | 1∙33 (1∙30, 1∙37) | <0∙001 |  | -- | -- | -- |
| Living space per capita (m^2^) | 41∙2 | 1∙36 (1∙32, 1∙40) |  | 47∙0 | 1∙31 (1∙26, 1∙36) | 0∙04 |  | 1∙27 (1∙22, 1∙33) | 1∙31 (1∙26, 1∙36) | 0∙23 |
| Urbanized areas (%) | 4∙9 | 1∙28 (1∙24, 1∙32) |  | 15∙8 | 1∙34 (1∙31, 1∙38) | <0∙001 |  | -- | -- | -- |
| Green areas (km^2^/100,000 persons) | 119∙4 | 1∙40 (1∙36, 1∙45) |  | 781∙9 | 1∙27 (1∙23, 1∙32) | <0∙001 |  | 1∙35 (1∙28, 1∙42) | 1∙27 (1∙23, 1∙32) | 0∙03 |
| Water bodies (km^2^/100,000 persons) | 0∙4 | 1∙36 (1∙32, 1∙40) |  | 5∙8 | 1∙34 (1∙30, 1∙37) | 0∙04 |  | 1∙31 (1∙26, 1∙35) | 1∙29 (1∙25, 1∙34) | 0∙17 |
| PM_2∙5_ (μg/m^3^) | 11∙4 | 1∙26 (1∙22, 1∙31) |  | 13∙1 | 1∙35 (1∙32, 1∙39) | <0∙001 |  | 1∙27 (1∙22, 1∙31) | 1∙37 (1∙31, 1∙42) | <0∙001 |
| O_3_ (μg/m^3^) | 43∙9 | 1∙40 (1∙36, 1∙44) |  | 51∙1 | 1∙25 (1∙21, 1∙29) | <0∙001 |  | 1∙39 (1∙34, 1∙45) | 1∙25 (1∙21, 1∙29) | <0∙001 |
| NO_2_ (μg/m^3^) | 11∙2 | 1∙24 (1∙20, 1∙28) |  | 18∙0 | 1∙37 (1∙33, 1∙40) | <0∙001 |  | 1∙24 (1∙20, 1∙28) | 1∙41 (1∙36, 1∙47) | <0∙001 |
| Temperature (°C) | 8∙9 | 1∙23 (1∙19, 1∙28) |  | 10∙0 | 1∙39 (1∙36, 1∙43) | <0∙001 |  | 1∙23 (1∙19, 1∙28) | 1∙38 (1∙33, 1∙44) | <0∙001 |

Heat effects at low and high levels of effect modifiers are represented as relative risks estimated from single- and two-predictor meta-regression with districts as the random effect.

CI=confidence interval, GDP=gross domestic product, NO_2_=nitrogen dioxide, O_3_=ozone, PM_2∙5_=particulate matter with a diameter of 2∙5 µm or less, RR=relative risk.

# Table S13. Heat effects on COPD mortality at low and high levels of effect modifiers (25th and 75th percentile of the modifier’s distribution) from single-predictor (left) and two-predictor (right, with adjustment for degree of urbanization) meta-regressions.

| **Characteristic** | **Single-predictor model** | | | | | |  | **Two-predictor model** | | |
| --- | --- | --- | --- | --- | --- | --- | --- | --- | --- | --- |
|  | **25th percentile** | |  | **75th percentile** | | ***p*-Wald** |  | **25th percentile** | **75th percentile** | ***p*-Wald** |
|  | **Value** | **RR (95% CI)** |  | **Value** | **RR (95% CI)** |  |  | **RR (95% CI)** | **RR (95% CI)** |  |
| Population aged≥65 years (%) | 18∙7 | 1∙27 (1∙22, 1∙33) |  | 21∙2 | 1∙25 (1∙20, 1∙31) | 0∙45 |  | 1∙25 (1∙18, 1∙31) | 1∙23 (1∙17, 1∙29) | 0∙56 |
| Foreigners (%) | 4∙4 | 1∙24 (1∙18, 1∙31) |  | 10∙5 | 1∙27 (1∙22, 1∙32) | 0∙25 |  | 1∙24 (1∙18, 1∙31) | 1∙23 (1∙16, 1∙31) | 0∙24 |
| Unemployment rate (%) | 5∙1 | 1∙25 (1∙19, 1∙32) |  | 9∙9 | 1∙27 (1∙22, 1∙32) | 0∙11 |  | 1∙24 (1∙18, 1∙31) | 1∙24 (1∙18, 1∙30) | 0∙13 |
| GDP per capita (€1,000) | 21∙6 | 1∙25 (1∙19, 1∙30) |  | 31∙5 | 1∙27 (1∙22, 1∙31) | 0∙18 |  | 1∙24 (1∙18, 1∙39) | 1∙24 (1∙18, 1∙30) | 0∙81 |
| Population density (persons/km^2^) | 123 | 1∙24 (1∙18, 1∙29) |  | 675 | 1∙26 (1∙21, 1∙31) | 0∙08 |  | -- | -- | -- |
| Living space per capita (m^2^) | 41∙2 | 1∙27 (1∙22, 1∙32) |  | 47∙0 | 1∙25 (1∙18, 1∙32) | 0∙52 |  | 1∙22 (1∙15, 1∙30) | 1∙25 (1∙18, 1∙32) | 0∙18 |
| Urbanized areas (%) | 4∙9 | 1∙23 (1∙17, 1∙29) |  | 15∙8 | 1∙26 (1∙22, 1∙31) | 0∙05 |  | -- | -- | -- |
| Green areas (km^2^/100,000 persons) | 119∙4 | 1∙29 (1∙23, 1∙35) |  | 781∙9 | 1∙23 (1∙17, 1∙30) | 0∙01 |  | 1∙26 (1∙17, 1∙35) | 1∙23 (1∙17, 1∙30) | 0∙24 |
| Water bodies (km^2^/100,000 persons) | 0∙4 | 1∙28 (1∙23, 1∙33) |  | 5∙8 | 1∙26 (1∙22, 1∙31) | 0∙33 |  | 1∙25 (1∙19, 1∙32) | 1∙24 (1∙19, 1∙30) | 0∙68 |
| PM_2∙5_ (μg/m^3^) | 11∙4 | 1∙21 (1∙15, 1∙27) |  | 13∙1 | 1∙27 (1∙22, 1∙31) | <0∙001 |  | 1∙21 (1∙15, 1∙27) | 1∙30 (1∙23, 1∙38) | 0∙01 |
| O_3_ (μg/m^3^) | 43∙9 | 1∙30 (1∙25, 1∙35) |  | 51∙1 | 1∙20 (1∙14, 1∙25) | <0∙001 |  | 1∙32 (1∙24, 1∙39) | 1∙20 (1∙14, 1∙26) | <0∙001 |
| NO_2_ (μg/m^3^) | 11∙2 | 1∙21 (1∙15, 1∙27) |  | 18∙0 | 1∙28 (1∙23, 1∙32) | <0∙001 |  | 1∙21 (1∙15, 1∙27) | 1∙30 (1∙23, 1∙38) | <0∙001 |
| Temperature (°C) | 8∙9 | 1∙21 (1∙15, 1∙27) |  | 10∙0 | 1∙29 (1∙24, 1∙34) | <0∙001 |  | 1∙21 (1∙15, 1∙27) | 1∙28 (1∙21, 1∙36) | <0∙001 |

Heat effects at low and high levels of effect modifiers are represented as relative risks estimated from single- and two-predictor meta-regression with districts as the random effect.

CI=confidence interval, GDP=gross domestic product, NO_2_=nitrogen dioxide, O_3_=ozone, PM_2∙5_=particulate matter with a diameter of 2∙5 µm or less, RR=relative risk.

# Table S14. Heat effects on pneumonia mortality at low and high levels of effect modifiers (25th and 75th percentile of the modifier’s distribution) from single-predictor (left) and two-predictor (right, with adjustment for degree of urbanization) meta-regressions.

| **Characteristic** | **Single-predictor model** | | | | | |  | **Two-predictor model** | | |
| --- | --- | --- | --- | --- | --- | --- | --- | --- | --- | --- |
|  | **25th percentile** | |  | **75th percentile** | | ***p*-Wald** |  | **25th percentile** | **75th percentile** | ***p*-Wald** |
|  | **Value** | **RR (95% CI)** |  | **Value** | **RR (95% CI)** |  |  | **RR (95% CI)** | **RR (95% CI)** |  |
| Population aged≥65 years (%) | 18∙7 | 1∙53 (1∙44, 1∙61) |  | 21∙2 | 1∙47 (1∙39, 1∙55) | 0∙25 |  | 1∙42 (1∙33, 1∙52) | 1∙39 (1∙31, 1∙48) | 0∙41 |
| Foreigners (%) | 4∙4 | 1∙36 (1∙28, 1∙44) |  | 10∙5 | 1∙57 (1∙49, 1∙64) | <0∙001 |  | 1∙35 (1∙27, 1∙44) | 1∙52 (1∙41, 1∙64) | 0∙01 |
| Unemployment rate (%) | 5∙1 | 1∙48 (1∙39, 1∙58) |  | 9∙9 | 1∙50 (1∙43, 1∙58) | 0∙75 |  | 1∙43 (1∙34, 1∙53) | 1∙39 (1∙31, 1∙47) | 0∙65 |
| GDP per capita (€1,000) | 21∙6 | 1∙43 (1∙35, 1∙51) |  | 31∙5 | 1∙51 (1∙44, 1∙58) | 0∙02 |  | 1∙39 (1∙31, 1∙47) | 1∙42 (1∙34, 1∙50) | 0∙63 |
| Population density (persons/km^2^) | 123 | 1∙39 (1∙32, 1∙48) |  | 675 | 1∙48 (1∙41, 1∙55) | <0∙001 |  | -- | -- | -- |
| Living space per capita (m^2^) | 41∙2 | 1∙54 (1∙46, 1∙62) |  | 47∙0 | 1∙43 (1∙34, 1∙52) | 0∙15 |  | 1∙38 (1∙28, 1∙49) | 1∙42 (1∙33, 1∙52) | 0∙84 |
| Urbanized areas (%) | 4∙9 | 1∙38 (1∙30, 1∙46) |  | 15∙8 | 1∙50 (1∙43, 1∙57) | <0∙001 |  | -- | -- | -- |
| Green areas (km^2^/100,000 persons) | 119∙4 | 1∙62 (1∙54, 1∙71) |  | 781∙9 | 1∙37 (1∙29, 1∙45) | <0∙001 |  | 1∙53 (1∙41, 1∙67) | 1∙37 (1∙29, 1∙45) | 0∙03 |
| Water bodies (km^2^/100,000 persons) | 0∙4 | 1∙54 (1∙46, 1∙61) |  | 5∙8 | 1∙50 (1∙43, 1∙57) | 0∙02 |  | 1∙43 (1∙34, 1∙52) | 1∙41 (1∙34, 1∙49) | 0∙11 |
| PM_2∙5_ (μg/m^3^) | 11∙4 | 1∙36 (1∙29, 1∙44) |  | 13∙1 | 1∙52 (1∙45, 1∙59) | <0∙001 |  | 1∙36 (1∙29, 1∙44) | 1∙52 (1∙42, 1∙62) | 0∙003 |
| O_3_ (μg/m^3^) | 43∙9 | 1∙60 (1∙53, 1∙68) |  | 51∙1 | 1∙35 (1∙28, 1∙43) | <0∙001 |  | 1∙55 (1∙45, 1∙66) | 1∙34 (1∙27, 1∙42) | <0∙001 |
| NO_2_ (μg/m^3^) | 11∙2 | 1∙32 (1∙25, 1∙40) |  | 18∙0 | 1∙55 (1∙49, 1∙62) | <0∙001 |  | 1∙32 (1∙25, 1∙40) | 1∙61 (1∙51, 1∙73) | <0∙001 |
| Temperature (°C) | 8∙9 | 1∙30 (1∙23, 1∙39) |  | 10∙0 | 1∙59 (1∙52, 1∙67) | <0∙001 |  | 1∙30 (1∙22, 1∙39) | 1∙55 (1∙45, 1∙66) | <0∙001 |

Heat effects at low and high levels of effect modifiers are represented as relative risks estimated from single- and two-predictor meta-regression with districts as the random effect.

CI=confidence interval, GDP=gross domestic product, NO_2_=nitrogen dioxide, O_3_=ozone, PM_2∙5_=particulate matter with a diameter of 2∙5 µm or less, RR=relative risk.


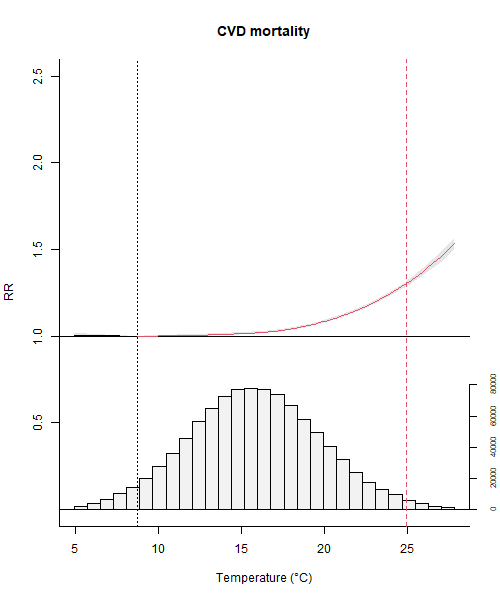

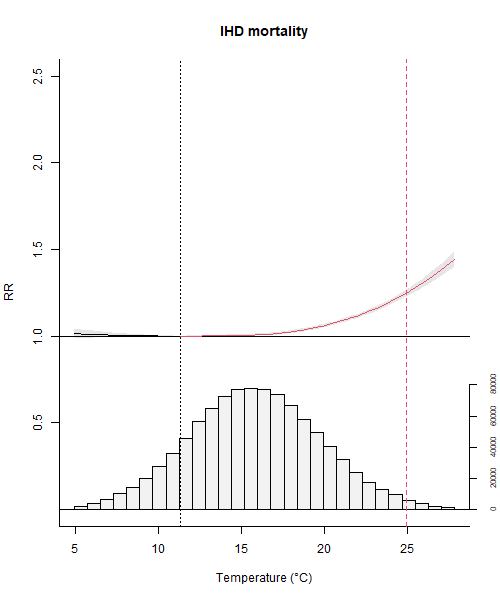

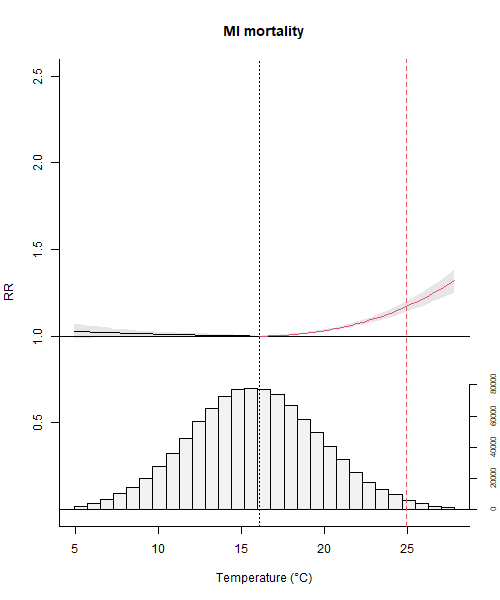

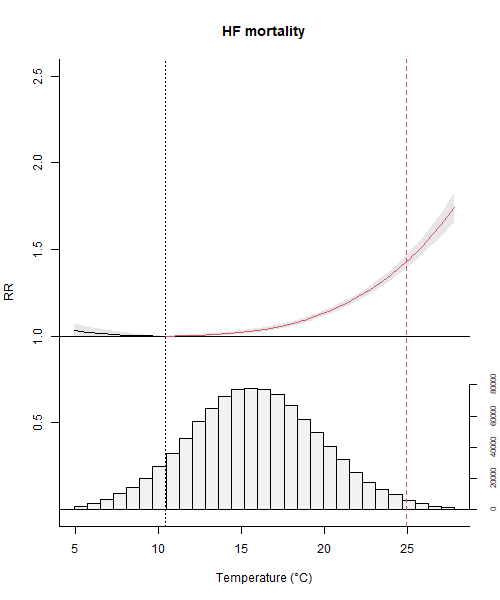

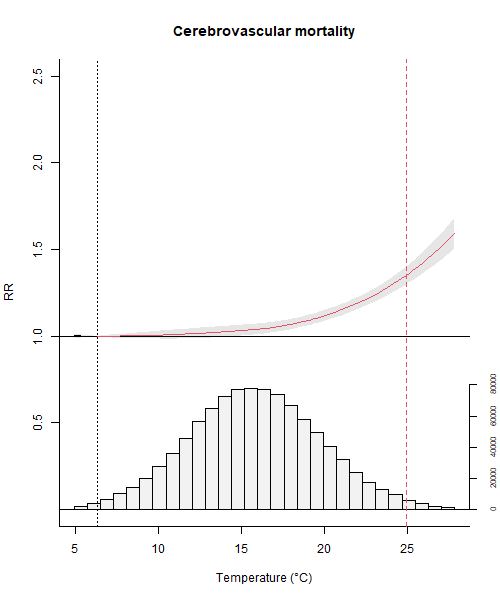

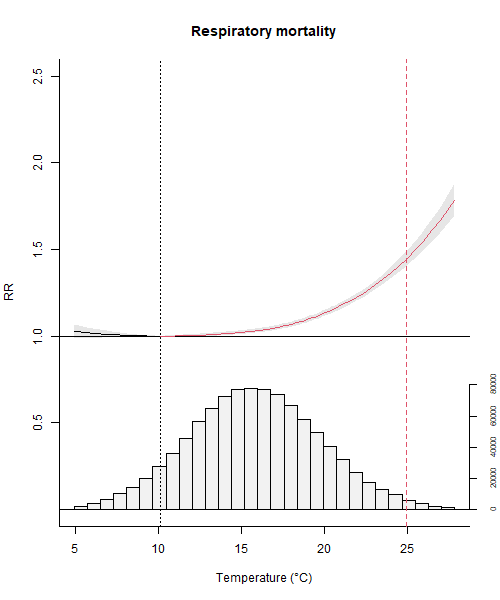

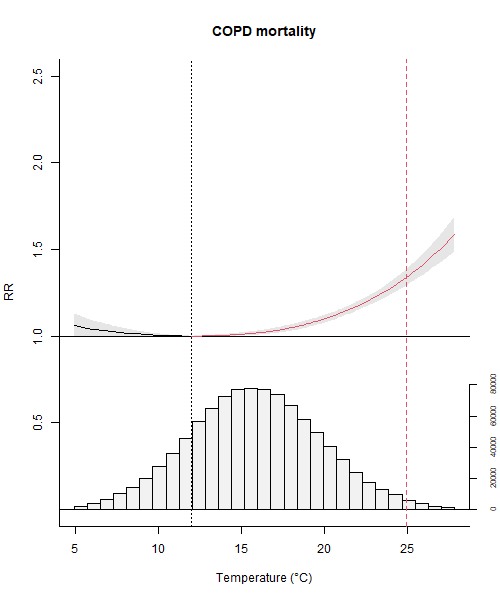

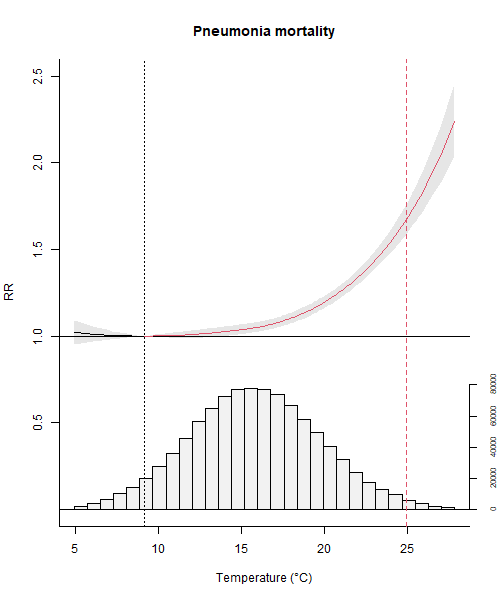


# Figure S1. Cumulative exposure-response relationships between air temperature and cause-specific cardiopulmonary mortality across Germany.

The black dotted line represents the minimum mortality temperature; the red dashed line represents the 99^th^ percentile of the temperature distribution.

COPD=chronic obstructive pulmonary disease, CVD=cardiovascular disease, HF=heart failure, IHD=ischemic heart disease, MI=myocardial infarction, RR=relative risk.


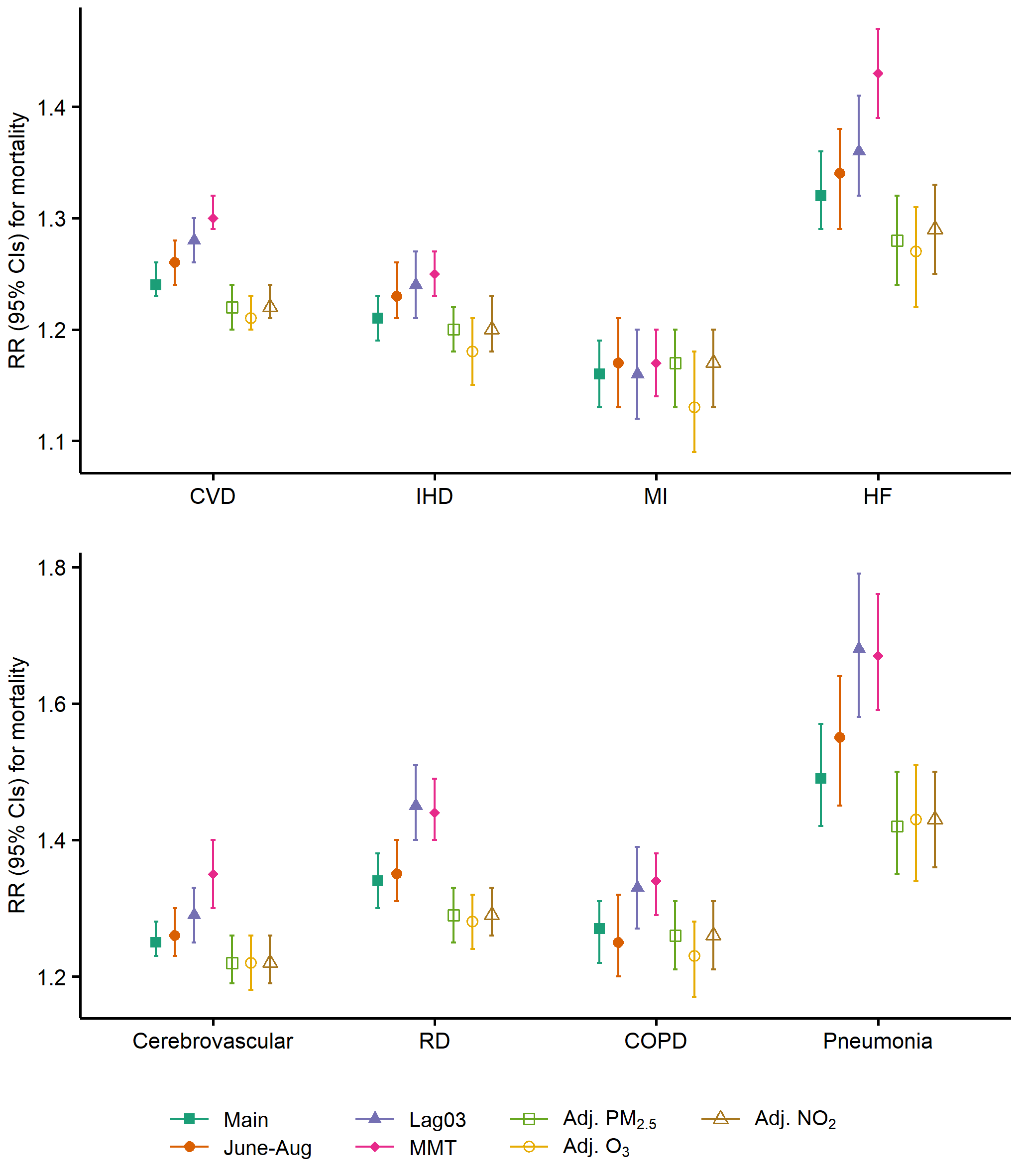


# Figure S2. Pooled relative risk (95% CI) of cause-specific cardiovascular and respiratory mortality for an increase in air temperature from the 75^th^ to the 99^th^ percentile in sensitivity analyses.

Sensitivity analyses: June-Aug: restricted warm season to June-August; Lag03: extended lag of heat effects to 0-3 days; MMT: heat effect estimates for an increase in air temperature from minimum mortality temperature (MMT) to the 99^th^ percentile; Adj. PM_2∙5_: adjusted for moving averages of PM_2∙5_ at lag 0-1 days; Adj. O_3_: adjusted for moving averages of O_3_ at lag 0-1 days; Adj. NO_2_: adjusted for moving averages of NO_2_ at lag 0-1 days.

CI=confidence interval, COPD=chronic obstructive pulmonary disease, CVD=cardiovascular disease, HF=heart failure, IHD=ischemic heart disease, MI=myocardial infarction, MMT=minimum mortality temperature, NO_2_=nitrogen dioxide, O_3_=ozone, PM_2∙5_=particulate matter with a diameter of 2∙5 µm or less, RD=respiratory disease, RR=relative risk.
